# Inhibition of autophagy as a novel treatment for neurofibromatosis type 1 tumors

**DOI:** 10.1002/1878-0261.13704

**Published:** 2024-08-11

**Authors:** Megan Stevens, Yuanli Wang, Stephanie J. Bouley, Torrey R. Mandigo, Aditi Sharma, Sonali Sengupta, Amy Housden, Norbert Perrimon, James A. Walker, Benjamin E. Housden

**Affiliations:** ^1^ Living Systems Institute University of Exeter UK; ^2^ Department of Clinical and Biomedical Science University of Exeter UK; ^3^ The First People's Hospital of Qinzhou China; ^4^ Center for Genomic Medicine Massachusetts General Hospital Boston MA USA; ^5^ Department of Genetics, Blavatnik Institute Harvard Medical School Boston MA USA; ^6^ Howard Hughes Medical Institute New York NY USA; ^7^ Cancer Program Broad Institute of MIT and Harvard Cambridge MA USA; ^8^ Department of Neurology, Massachusetts General Hospital Harvard Medical School Boston MA USA

**Keywords:** autophagy, *Drosophila*, drug repurposing, neurofibromatosis type 1, synthetic lethality

## Abstract

Neurofibromatosis type 1 (NF1) is a genetic disorder caused by mutation of the *NF1* gene that is associated with various symptoms, including the formation of benign tumors, called neurofibromas, within nerves. Drug treatments are currently limited. The mitogen‐activated protein kinase kinase (MEK) inhibitor selumetinib is used for a subset of plexiform neurofibromas (PNs) but is not always effective and can cause side effects. Therefore, there is a clear need to discover new drugs to target *NF1*‐deficient tumor cells. Using a *Drosophila* cell model of NF1, we performed synthetic lethal screens to identify novel drug targets. We identified 54 gene candidates, which were validated with variable dose analysis as a secondary screen. Pathways associated with five candidates could be targeted using existing drugs. Among these, chloroquine (CQ) and bafilomycin A1, known to target the autophagy pathway, showed the greatest potential for selectively killing *NF1*‐deficient *Drosophila* cells. When further investigating autophagy‐related genes, we found that 14 out of 30 genes tested had a synthetic lethal interaction with *NF1*. These 14 genes are involved in multiple aspects of the autophagy pathway and can be targeted with additional drugs that mediate the autophagy pathway, although CQ was the most effective. The lethal effect of autophagy inhibitors was conserved in a panel of human *NF1*‐deficient Schwann cell lines, highlighting their translational potential. The effect of CQ was also conserved in a *Drosophila NF1 in vivo* model and in a xenografted *NF1*‐deficient tumor cell line grown in mice, with CQ treatment resulting in a more significant reduction in tumor growth than selumetinib treatment. Furthermore, combined treatment with CQ and selumetinib resulted in a further reduction in *NF1*‐deficient cell viability. In conclusion, *NF1*‐deficient cells are vulnerable to disruption of the autophagy pathway. This pathway represents a promising target for the treatment of *NF1*‐associated tumors, and we identified CQ as a candidate drug for the treatment of *NF1* tumors.

## Graphical abstract

We used synthetic lethal screens to find new approaches to treat neurofibromatosis type 1 (NF1) tumors. Inhibition of autophagy was identified as a robust method to selectively kill NF1‐deficient cells with minimal effects on healthy cells. Following assessment of a range of autophagy inhibitors, we determined that chloroquine has strong potential for repurposing to treat NF1‐associated tumors.

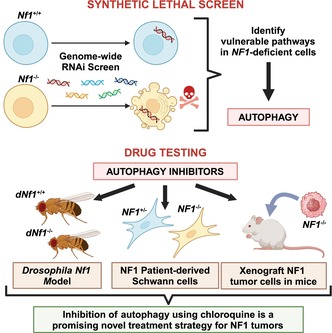

**Abbreviations**
ATP: adenosine triphosphate
CDI: coefficient of drug interaction
CQ: chloroquine
CRISPR: clustered regularly interspaced short palindromic repeats
DAPI: 4′,6‐diamidino‐2‐phenylindole
ERK: extracellular signal‐regulated kinase
hTERT: human telomerase reverse transcriptase
MEK: mitogen‐activated protein kinase kinase
MPNST: malignant peripheral nerve sheath tumor
NF1: neurofibromatosis type 1
PAM: protospacer adjacent motif
PI: propidium iodide
sgRNA: single guide RNA
shRNA: short hairpin RNA
UAS: upstream activating sequence
VDA: variable dose analysis
WT: wild‐type

## 1 Introduction

Neurofibromatosis type 1 (NF1) is an autosomal dominant genetic disorder that affects approximately 1 in 2700 live births [1, 2]. NF1 is characterized by highly variable symptoms, including benign, but often disfiguring, peripheral nerve‐associated tumors known as neurofibromas (plexiform and cutaneous) found pervasively in almost all patients, as well as cancerous tumors, such as malignant peripheral nerve sheath tumors (MPNSTs), which are usually fatal [3]. NF1 patients have reduced life expectancy by around 15 years, partly due to the higher prevalence of vascular defects and cancer [4].

NF1 is caused by mutations in the *NF1* gene, causing loss of function of neurofibromin, a 320 kDa protein, which is a RAS GTPase Activating Protein (RASGAP) for H‐, K‐, N‐RAS and R‐RAS1, 2, and 3 [5, 6, 7, 8]. RASGAPs promote the conversion of the active RAS‐GTP form into the inactive RAS‐GDP form by stimulating RAS‐GTP hydrolysis. Loss of neurofibromin therefore causes disruption of the RAS signaling pathway, with the best characterized downstream effectors being the RAF/MEK/ERK and PI3K/AKT/mTOR pathways [9]. Disruption of RAS signaling is thought to be the main cause of NF1 symptoms. However, it remains unclear which pathways downstream of RAS are responsible for disease development and progression. In addition, it is likely that crosstalk occurs between the pathways downstream of RAS, making the mechanisms of disease even more complex. Inhibition of RAS pathway components such as MEK or ERK is therefore a therapeutic option for NF1 patients. However, the robust regulation of RAS [9] may complicate the outcomes of therapy and may explain why, despite considerable study, no broadly effective treatment for RAS‐driven cancers has yet been developed. Furthermore, long‐term inhibition of the RAS pathway is likely not an optimal treatment strategy for the serious but non‐life‐threatening symptoms of NF1, especially in children.

Currently, there are limited therapies for any NF1‐associated tumors. The only available drug is the MEK inhibitor selumetinib, which was approved by the FDA for use in a subset of inoperable pediatric plexiform neurofibromas (PNs) in April 2020 [10]. However, not all tumors were responsive to treatment and serious side effects can be associated with MEK inhibitor use [10, 11, 12, 13, 14]. Therefore, there is a clear clinical need to discover new drugs that specifically target *NF1*‐deficient tumor cells either alone or in combination with selumetinib.

One approach to identify candidate drug targets for tumorigenic diseases is to use synthetic lethal interaction screens. Synthetic lethal interactions are a type of genetic interaction in which inhibition of either of two genes alone is viable, but the combined inhibition of both genes is inviable. When one of these genes is mutated in tumor cells, such interactions can be exploited to kill those cells exclusively by targeting the synthetic lethal partner gene using a drug [15, 16]. This approach is attractive because treatment is expected to be lethal to tumor cells but have no effect on wild‐type, healthy cells.

Despite long‐term interest in the use of synthetic lethality as a therapeutic strategy to treat tumors, few drugs have successfully progressed to clinical use. A major factor preventing successful development of treatments against synthetic lethal interactions is a lack of consistency between interactions identified in different genetic backgrounds [17]. This results in a lack of translation of candidate targets between model systems. To overcome this limitation, our approach makes use of *Drosophila* cells to initially identify synthetic lethal interactions with genes mutated in tumors. The conservation of candidate interactions can then be assessed in a range of other model systems, including human cells, providing a filter to remove interactions that are specific to a single model system. This approach has previously proved successful, leading to the discovery of mizoribine and palbociclib as promising candidates for the treatment of tuberous sclerosis complex (TSC) and Von Hippel–Lindau (VHL)‐linked cancers, respectively [18, 19, 20]. In both cases, hits from *Drosophila* synthetic lethal screens were validated with a high success rate in both human cells and mouse models.

Given the previous success of using the *Drosophila* approach, we have applied this method to identify candidate drug targets to treat *NF1*‐deficient tumors. Here, we describe the generation of a *dNF1* null mutant *Drosophila* cell line using CRISPR and its use in synthetic lethal screens to identify candidate drug targets to specifically kill NF1‐associated tumor cells. We find *dNF1*‐deficient cells are vulnerable to inhibition of autophagy. Importantly, we show that this selective effect can be reproduced with multiple drugs known to modulate autophagy and in several NF1 patient‐matched tumor‐derived cell lines, as well as *Drosophila in vivo* NF1 models and xenografts of *NF1‐*deficient tumor cell lines in mice, indicating that these repurposed drugs may have promise for the treatment of NF1‐associated tumors in the future. Finally, we show that combined treatment with CQ or bafilomycin A1 and selumetinib results in increased selective killing of *NF1*‐deficient cells, indicating the potential for combinatorial therapy.

## 2 Materials and methods

### 2.1 Cell culture

*Drosophila* Schneider (S2R+) cells, both WT and dNF1‐KO, were cultured at 25 °C in Schneider's media (Gibco: ThermoFisher Scientific Waltham, MA USA) containing 1% antibiotic (Gibco: ThermoFisher Scientific Waltham, MA USA) and 10% fetal bovine serum (Gibco: ThermoFisher Scientific Waltham, MA USA). Human cell lines used include: *NF1* ^*+/−*^ and *NF1* ^*−/−*^ immortalized human Schwann cell (SC) lines, derived from the ipn02.3 2λ (hTERT ipn02.3 2λ CRL‐3392) cell line using CRISPR/Cas9 gene editing as described below; two immortalized human SCs derived from PNs from NF1 patients [21], which included ipnNF95.11C (hTERT NF1 ipnNF95.11c, RRID: CVCL_UI69; *NF* ^*+/−*^) and ipNF95.11b ‘C’ (hTERT NF1 ipNF95.11b C, RRID: CVCL_UI67; *NF1* ^*−/−*^) cells (germline *NF1* mutation: c.1756delACTA), and ipnNF09.4 (hTERT NF1 ipnNF09.4, RRID: CVCL_UI73; *NF1* ^*+/−*^) and ipNF05.5 (hTERT NF1 ipNF05.5, RRID: CVCL_UI71; *NF1* ^*−/−*^) cells (germline *NF1* mutation: c.3456_3457insA). All human cell lines were cultured at 37 °C in 5% CO_2_ in DMEM media (Merck, Rahway, NJ USA) containing 1% antibiotic (Gibco: ThermoFisher Scientific Waltham, MA USA) and 10% fetal bovine serum (Gibco: ThermoFisher Scientific Waltham, MA USA). For ipnNF09.4 and ipNF05.5 cells, the media was also supplemented with 50 ng·mL^−1^ neuregulin‐1 (NRG‐1) (Sigma, Burlington, MA USA).

All cell lines have been authenticated in the past 3 years regarding the expression of NF1 using Western blotting and an antibody specific for NF1 (Abcam, Cambridge, United Kingdom; ab238142). Western blotting confirmed that NF1‐deficient cells did not express the NF1 protein. In addition, all experiments were performed in mycoplasma‐free cells.

### 2.2 Generation of dNF1‐KO *Drosophila* cells

**Fig. 1:**
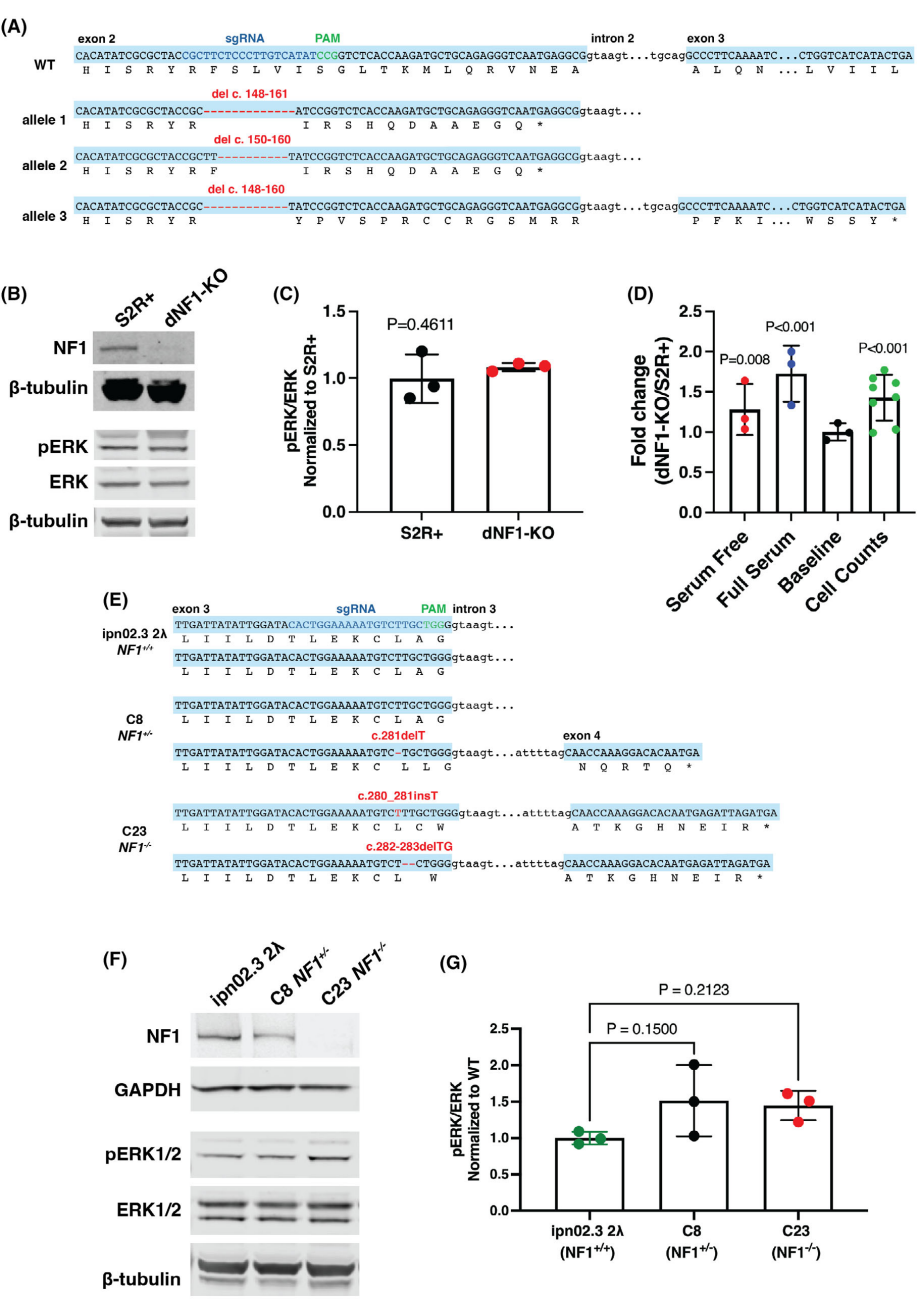
Generation and characterization of NF1‐deficient *Drosophila* and human Schwann cells using CRISPR. (A) *Drosophila* S2R+ cells were transfected with Cas9 and sgRNA designed to target a double‐stranded break in exon 2 of *dNF1*. Molecular analysis of the dNF1‐KO line revealed deletions of 14, 11 and 13 bp in three alleles (S2R+ cells are aneuploid). The position of the guide RNA is shown in blue and the protospacer adjacent motif (PAM) site in green. Predicted effect of deletions on the amino acids in each allele are shown, each resulting in premature termination. (B) Representative image of western blot showing loss of neurofibromin in dNF1‐KO cells compared to wild‐type (WT) S2R+ cells (two replicates performed) and pERK and total ERK levels in S2R+ and dNF1‐KO cells under normal culturing conditions (10% serum). (C) Quantification of pERK/ERK ratio in S2R+ and dNF1‐KO cells (in 10% serum) shows no significant change (from triplicate experiments). Error bars indicate standard deviation; *P*‐value was calculated using a two‐tailed, unpaired *t*‐test. (D) Characterization of dNF1‐KO cell size and proliferation rate as assessed using CellTiter‐Glo assays or cell counts (number of replicates is indicated by the colored circles on each bar, error bars indicate standard deviation, *P* values determined using unpaired (except for cell counts, which were paired), two‐tailed *t*‐tests). ‘Baseline’ indicates a comparison of ATP levels with no incubation for growth. ‘Cell counts’ indicates that cell numbers were counted following 72 h of growth from equal initial cell populations. ‘Serum free’ and ‘Full serum’ indicate ATP measurements following 72 h of incubation for growth from initially equal cell populations. (E) CRISPR/Cas9 was used to target exon 3 of *NF1* in a telomerase‐immortalized human Schwann cell line (hTERT ipn02.3 2λ) [21] to generate isogenic NF1 knock out (NF1^−/+^ and NF1^−/−^) cell lines. Line C8 has a heterozygous 1 bp deletion (c.281delT) and line C23 is a transheterozygous combination of 1 bp insertion (c.280‐281insT) and 2 bp deletions (c.282–283 delTG). Position of the guide RNA (NF1‐sg2) is shown in blue and the PAM site in green. (F) Western blot analysis of cell lines showing reduction (C8) and absence (C23) of neurofibromin compared to the wild‐type ipn02.3 2λ progenitor Schwann cell line. pERK and total ERK levels were assessed in cells grown in 10% serum. The image is of a representative blot from three replicates (G) Quantification of pERK/ERK ratios shows no significant change between wild‐type and C8 or C23 cells (from triplicate experiments). Error bars represent the standard deviation and *P*‐values were calculated using a one‐way ANOVA with multiple comparisons.

The dNF1‐KO cell line was generated using methods described previously [20, 22]. WT S2R+ cells were co‐transfected with the pl018 plasmid to express Cas9 and the sgRNA (CGCTTCTCCCTTGTCATATC) and pAct‐GFP plasmid to mark transfected cells. Five days after transfection, individual cells with the highest 15% GFP signal were isolated and seeded into wells of 96‐well plates using FACS. Single cells were cultured using conditioned media for approximately 3 weeks. DNA was then extracted from each candidate cell population and assessed using HRMA to identify those carrying mutations at the sgRNA target site. Positive candidates were then sequenced to confirm frame‐shift mutations were present in each *NF1* allele (Fig. 1A).

### 2.3 Generation of *NF1* ‐deficient human Schwann cell lines using CRISPR‐Cas9

The wild‐type human hTERT‐immortalized Schwann cell line (hTERT ipn02.3 2λ) was a gift from Dr. Peggy Wallace, University of Florida [21]. CRISPR‐Cas9 genome editing was performed using NF1‐sg2 (ACACTGGAAAAATGTCTTGC), a sgRNA targeting human *NF1* exon 3 in the lentiCRISPR v1 plasmid, which was a gift from Dr. Feng Zhang (Broad Institute) [23]. hTERT ipn02.3 2λ cells were transfected by electroporation using the Amaxa Basic Nucleofector Kit for Primary Mammalian Fibroblasts (Lonza, Basel, Switzerland) and the program U‐023, according to the manufacturer's instructions. After 48 h of selection on puromycin (1 μg·mL^−1^) in DMEM/10% FBS, cells were re‐plated in to allow single clone isolation. Genotyping of selected single clones was performed using PCR using primers NF1‐exon3‐FOR (CCCCAATTCAAGATTCTGGT) and NF1‐exon3‐REV (ATCGCACTCTCCCACAACTC). PCR products were treated with exoSAP (Affymetrix, Santa Clara, CA USA) and sequenced using NF1‐exon3‐SEQ (TGCCATTTCTGTTTGCCTTA).

### 2.4 Synthetic lethal screen

Screens were performed as described previously [22]. Specifically, we used wild‐type S2R+ or dNF1‐KO cells and the genome‐wide RNAi library from the Sheffield RNAi Screening Facility. In total, 10 000 cells were seeded into each library well in 10 μL serum‐free Schneider's *Drosophila* media. Plates were then incubated at room temperature for 45 min before the addition of 35 μL media with 10% FBS. Liquid handling was performed using a Multidrop Combi. Libraries were incubated at 25 °C for 5 days before CellTiter‐Glo assays (Promega, Madison, WI USA) were performed. ATP levels were assessed using luminescence from the CellTiter‐Glo assays measured with a TECAN Infinite M200 Pro plate reader. Screens were performed in triplicate in each cell line (Fig. S1).

Data were analyzed by first normalizing all values to the median of each row and column of the library plate to allow direct comparison between plates. *Z*‐scores were then calculated for each RNAi reagent using the average and standard deviation of each replicate screen and correlation between replicates was used to assess the quality of screen results. Reagents were considered hits if the *Z*‐score in at least 2/3 of replicates was below −1.5 in dNF1‐KO cells and above −1.5 in S2R+ cells. We note that some assay plates were affected by position effects; these were identified manually and removed from the analysis prior to correlation analysis.

Functional gene groups were determined using the in‐built clustering tool in the STRING database to group genes. Functions of each group were determined manually by searching for associated GO terms or assessing annotated functions of human orthologs in cases where the *Drosophila* gene was not sufficiently annotated.

### 2.5 Variable dose analysis (VDA)

VDA is an RNAi‐based method in which each cell within a population receives a different dose of shRNA [24, 25]. The relative knockdown efficiency of each cell is then measured with a fluorescent reporter. On the day of transfection S2R+ and dNF1‐KO cells were plated at 1 × 10^4^ cell/100 μL culture media, per well of a 96‐well plate. Cells were incubated at 25 °C for 40 min to allow adhesion. Cells were then transfected with 40 ng actin‐GFP and 160 ng shRNA expression plasmid using 0.6 μL FuGENE® HD transfection reagent in a total volume of 10 μL. We used a positive (*thread*, an apoptosis inhibitor that induces cell death when inhibited) and negative (*white*, known to have no viability effect in these cells) shRNA on each plate for normalization of data. Plates were then sealed and incubated for 4 days at 25 °C in a humidifying chamber.

Flow cytometry was used to identify GFP‐positive cells (transfection efficiency). The area under an inverted cumulative distribution curve was used as a readout of relative viability, normalized to the positive and negative control. More detailed protocol and data analysis information can be found in Sierzputowska *et al*. [25].

### 2.6 CellTiter‐Glo assays

The CellTiter‐Glo assay (Promega; G7570) is a luminescent viability assay based on the quantification of ATP, which is an indicator of metabolically active cells. All cells were plated in white 384‐well plates at a seeding density of 5 × 10^3^ cells/25 μL culture media in either complete or serum‐free media. S2R+ cells were left to adhere for 40 min at 25 °C and human cells were left to adhere for 4 h at 37 °C in 5% CO_2_ before treatment. Cells were treated with 250 nL per well of each drug (CQ, bafilomycin A1, and selumetinib) in PBS at varying concentrations in replicates of 5–8 using the Mosquito LV Genomics (SPT Labtech, Melbourn, Cambridgeshire, United Kingdom) and incubated for 48 h. The CellTiter‐Glo assay was then performed according to the manufacturer's instructions, and luminescence was measured using a plate reader (TECAN Infinite M200 Pro, Männedorf, Switzerland).

### 2.7 Cell count assays

Human cells were seeded in 24‐well plates at a density of 2.5 × 10^4^ cells per well in complete culture media and left to adhere for 4–6 h at 37 °C in 5% CO_2_ before treatment. The cells were subsequently treated with CQ, bafilomycin A1, or selumetinib in PBS in serum‐free media. Counts were performed 24 and 48 h later, with trypan blue used to exclude dead cells.

### 2.8 Autophagy assay and caspase assay

An autophagy assay kit (Abcam; ab139484) was used to measure autophagic vacuoles in live cells using a cationic amphiphilic tracer (CAT) dye that selectively labels autophagic vacuoles. This dye has been optimized to ensure the minimal staining of lysosomes and results in bright fluorescence upon incorporation into pre‐autophagosomes, autophagosomes, and autophagolysosomes. All cells were plated in 96‐well plates in either complete or serum‐free media. S2R+ cells were left to adhere for 40 min at 25 °C and human cells were left to adhere for 4 h at 37 °C in 5% CO_2_ before treatment. Cells were treated with 10 μm per well of CQ in PBS at varying concentrations and incubated for 4 h to block autophagosome fusion with lysosomes. Cells were then washed three times with PBS and fluorescence intensity was measured using a plate reader at Ex/Em 480/530 nm (TECAN Infinite M200 Pro) to provide a measure of autophagic flux. The higher the fluorescence intensity, the higher the buildup of autophagosomes, and therefore, the higher the rate of autophagic flux. Measurements in *NF1*‐deficient cells were normalized to the average of control cells.

Caspase activity was assessed using the generic caspase activity assay kit (Abcam; ab112130). All cells were plated in 96‐well plates in either complete or serum‐free media. S2R+ cells were left to adhere for 40 min at 25 °C and human cells were left to adhere for 4 h at 37 °C in 5% CO_2_ before treatment. Cells were subsequently treated with CQ or bafilomycin in serum‐free media for 48 h. The cell permeable and non‐toxic TF2‐VAD‐FMK fluorescent indicator, which irreversible binds to caspase‐1, ‐3, ‐4, ‐5, ‐6, ‐7, ‐8, and ‐9 in apoptotic cells, was then added to the cells for 4 h at room temperature. Cells were subsequently washed three times in PBS and the fluorescent intensity was measured using a plate reader at Ex/Em 490/525 nm (TECAN Infinite M200 Pro). Measurements in *NF1*‐deficient cells were normalized to the average of control cells.

In both the autophagy and caspase assay, autophagy/caspase fluorescence was normalized to Hoechst fluorescence in each well (Ex/Em 370/485 nm) to account for changes in cell density.

### 2.9 Annexin V and PI staining

The Annexin V‐FITC apoptosis kit (Abcam; ab14085) was used to detect apoptosis by staining phosphatidylserine molecules that have translocated to the outside of the cell membrane. Cells were co‐stained with propidium iodide (PI) to detect dead cells in the population. S2R+ cells were left to adhere for 40 min at 25 °C before treatment. Cells were treated with 1 μL per well of each drug (CQ, bafilomycin A1) in PBS at varying concentrations, and incubated for 48 h. The cells were subsequently incubated in Annexin‐V‐FITC and PI assay for 5 min at room temperature in the dark, and fluorescence intensity was measured using flow cytometry (Annexin V‐FITC Ex/Em 488/525 nm and PI 561/585 nm).

### 2.10 Western blotting

Lysates from *Drosophila* S2R+ cells, human immortalized Schwann cells, and homogenized xenograft tissue for western blotting were prepared in RIPA buffer supplemented with protease inhibitors (Complete Protease Inhibitor Cocktail, Sigma) and phosphatase inhibitors (NaF/Na_3_VO_4_/β‐glycerophosphate). Lysates were analyzed on NuPAGE 3–8% Tris‐Acetate gels to assess neurofibromin levels and on NuPAGE 4–12% Bis‐Tris protein gels to assess pERK/ERK levels; lysates for testing the expression of pERK/ERK, pAKT/AKT, PARP, and LC3B were resolved on stain‐free TGX gels (4–15%; BIO‐RAD, Hercules, CA USA). Gels were transferred to nitrocellulose membranes and blots were processed using the Odyssey CLx protocol (LI‐COR, Lincoln, NE USA). Similar to previous publications [26], neurofibromin expression in adult flies was assayed using immunoprecipitation (IP) followed by western blotting. Lysates of heads from 2‐ to 3‐day old adult flies were prepared in IP buffer (50 mm Tris–HCl pH 7.5, 125 mm NaCl, 1.5 mm MgCl_2_, 0.2% NP‐40, 5% glycerol) with addition of protease inhibitors and phosphatase inhibitors (as above). IPs were performed with 400 μg of total protein incubated in 1 mL IP buffer with 200 μL of mAb21 anti‐*Drosophila* Nf1 monoclonal antibody supernatant [27] at 4 °C for 1 h. Protein A‐ and Protein G‐Sepharose beads (1 : 1, Sigma) were used to precipitate Immunocomplexes at 4 °C for 1 h. Beads were then washed three times with IP buffer, denatured using 2× SDS sample buffer and analyzed on NuPAGE 3–8% Tris‐Acetate gels to assess neurofibromin levels. Lysates used in the immunoprecipitations were also blotted for β‐tubulin. Antibodies used: anti‐*Drosophila* neurofibromin (mouse, ascites purified mAb21 and mAb30, 1 : 500 each), anti‐human neurofibromin (rabbit, Infixion r07E, San Diego, CA USA, 1 : 1000), anti‐phospho‐ERK (mouse, Sigma M8159, 1 : 2500), total ERK (rabbit, CST 9102, Danvers, MA USA, 1 : 1000), anti‐β‐tubulin (mouse, Developmental Studies Hybridoma Bank E7, Iowa City, IA USA, 1 : 10 000), GAPDH (rabbit, Proteintech 10 494‐1‐AP, San Diego, CA USA, 1 : 15 000), anti‐PARP (rabbit, Cell Signaling 952, 1 : 1000), and anti‐LC3B (rabbit, Cell Signaling 2775, 1 : 1000; CST Danvers, MA USA). Secondary antibodies used were anti‐mouse Alexa Fluor Plus 800 (goat, Invitrogen, A32730, 1 : 10 000) and anti‐rabbit Alexa Fluor Plus 680 (goat, Invitrogen, Waltham, MA USA, A21109, 1 : 10 000). In cases where TGX gels were used, protein of interest expression was normalized to the total protein on the membrane, which was imaged using the BIORAD EZ Imager.

### 2.11 *Drosophila* husbandry and stocks

Flies were cultured on cornmeal/agar food medium according to standard protocol and housed at 25 °C.

The *Nf1* ^*C1*^ mutant fly line was generated by CRISPR/Cas9 [28] using the sgRNA line: *y* [1] sc[*] v [1] *sev* [21]; *P{y[+t7.7] v[+t1.8] = TKO.GS01796}attP40* (GS01796 sgRNA sequence: CGCTTCTCCCTTGTCATATC) and a germline source of Cas9: *y* [1] M{w[+mC] = nos‐Cas9.P}ZH‐2A w[*] (Bloomington Stock #54591). The *UAS‐dNf1* transgene encodes full‐length *dNf1* cDNA corresponding to the RF isoform with addition of introns 9 and 10 and was previously published [29]. The RNAi and landing site control line were obtained from the Vienna *Drosophila* RNAi Center: *dNf1 RNAi* line: P{KK101909}VIE‐260B (VDRC #109637) and VIE‐260B (VDRC #60100). *UAS‐Dicer2* was included to potentiate the RNAi effect [30]: *P{w[+mC] = UAS‐Dcr‐2.D}10* (Bloomington: 24651). Male flies were used for all experiments.

### 2.12 Assessing drug sensitivity in flies

Flies were raised on standard fly food and kept at 25 °C. For testing drugs, adult flies were transferred to Formula 4–24 Instant *Drosophila* Medium (Carolina Biological, Burlington, NC USA) prepared using water (control) or chloroquine (chloroquine diphosphate; Sigma C6628) in water. For each genotype, three replicates of 20 flies were added to each condition and kept at 25 °C. Flies were monitored periodically to assess lethality. Survival was reported as the percent of flies still alive at each time point.

### 2.13 NF1 tumor xenografts and toxicity assays

All experiments were conducted in accordance with UK legislation and with local ethics committee approval (University of Exeter AWERB), under project license P983E59F3. Mice were housed in individually vented cages (Techniplast), with 3–4 mice per cage. They were housed in a sterile room with a 12 h/12 h light/dark cycle, 21 °C, and 45–55% humidity. Mice were handled under a laminar airflow hood and underwent procedures in a sterile surgical room. Mice were 6–8 weeks of age at the beginning of each study. Two million ST88‐14 (RRID: CVCL_8916) cells mixed 1 : 1 with Matrigel (ThermoFisher Scientific, Waltham, MA USA) were injected subcutaneously into the right flank of male CD‐1 nude mice (Athymic Nude Mice obtained from Charles River (Crl:NU(NCr)‐Foxn1nu)). Tumors were measured three times weekly with a caliper, and the tumor volume was calculated as follows: [(length + width)/2] × length × width. Once the tumor diameter began to increase over two separate measurements, mice were intraperitoneally injected with either saline or CQ (50 mg·kg^−1^ in saline) or received selumetinib (25 mg·kg^−1^ in saline) via oral gavage three times per week (*n* = 6 mice per group). Mice were culled by cervical dislocation (Schedule 1) when the control tumor sizes reached the allowed endpoint (12 mm in diameter), and tumors were dissected. Tumors were flash frozen for further analysis.

Toxicity studies were performed using CD‐1 mice and C57BL/6J Inbred Mice (JAX® Mice Strain) obtained from Charles River.

## 3 Results

### 3.1 Generation of isogenic *NF1* ‐deficient *Drosophila* and human Schwann cell models using CRISPR/Cas9 gene editing

Our previous studies have demonstrated the potential of using cross‐species genetic screens to identify candidate therapeutic targets for human disease [18, 19, 20, 24]. The *NF1* gene is well conserved between *Drosophila* and humans with 68% identity at the amino acid level (Fig. S2). To use the same approach to find new targets for the treatment of NF1‐associated tumors, we first used CRISPR gene editing to generate indel mutations in *dNF1* in *Drosophila* S2R+ cells. Sequencing was used to confirm that the induced frame‐shift mutations resulted in null *dNF1* alleles (Fig. 1A). Of note, S2R+ cells are aneuploid [31], and our sequencing results suggest that they have three copies of the *dNF1* gene.

The resulting dNF1‐KO S2R+ cell line (hereafter called dNF1‐KO) was characterized by assessing the expression of neurofibromin using western blots. We found no detectable signal in the dNF1‐KO line compared to parental wild‐type (WT) S2R+ cells (Fig. 1B, Fig. S3A). However, we found no significant increase in pERK or pAKT levels in dNF1‐KO cells under normal culturing conditions or serum starved conditions (Fig. 1B,C, Fig. S3B,E). This is consistent with the results of previous studies using RNAi to knock down *dNF1* in S2R+ cells, which suggest that the lack of effect is due to redundancy with the Gap1 protein [32, 33]. Given that neurofibromin is a negative regulator of RAS, we assessed the growth and proliferative phenotypes of dNF1‐KO cells compared to S2R+ cells. Consistent with deregulation of a mitogenic pathway, dNF1‐KO cells showed an increased rate of growth as measured using CellTiter‐Glo assays to assess total ATP levels in the population. This effect was observed in both the presence and absence of serum in the culture media (Fig. 1D), indicating that culture growth is both accelerated in the absence of *dNF1* and is decoupled from upstream growth factor signaling pathways. To determine whether this increase in culture growth was due to increased proliferation, increased cell growth, or both, we performed cell counts following culture in full serum and CellTiter‐Glo assays on normalized numbers of cells from each genotype (baseline readings). Cell counts for dNF1‐KO showed an increase in cell numbers following culture compared to S2R+ cells, and the ‘baseline’ CellTiter‐Glo showed no difference (Fig. 1D). This suggests that the difference in culture growth is primarily due to increased cell proliferation rather than an increase in the cell size or ATP content of the cells. Together, these results indicate that the dNF1‐KO line represents a novel *dNF1* null mutant cell model, with properties consistent with known effects of NF1 loss.

We also utilized CRISPR/Cas9 gene editing to generate indel mutations within exon 3 of *NF1* in wild‐type immortalized human Schwann cells (ipn02.3 2λ). Sequencing of single cell clones was used to confirm out‐of‐frame deletions in one or both *NF1* alleles, resulting in *NF1*‐deficient cell lines (Fig. 1E). The resulting heterozygous (C8) and *NF1* null (C23) cell lines were characterized by assessing neurofibromin expression using western blots (Fig. 1F). Consistent with what we observed in dNF1‐KO cells, there was no significant increase in pERK or pAKT expression under normal or serum starved culturing conditions (Fig. 1F,G, Fig. S1C,F). Our results suggest that these otherwise isogenic human cell lines are an appropriate model to validate any results obtained in our dNF1‐KO cell lines.

### 3.2 Mapping synthetic lethal interactions in dNF1‐KO cells using a genome‐wide RNAi screen

We used a genome‐wide dsRNA library to screen in both S2R+ and dNF1‐KO cells for synthetic lethal interactions (Fig. S1). The screen was performed by treating dNF1‐KO and S2R+ cells with dsRNA reagents, incubating under standard culture conditions for 5 days before assessing cell viability using CellTiter‐glo assays (see Materials and Methods section for details). Correlation coefficients ranged between 0.9 and 0.99 (average 0.93) for control wells and between 0.55 and 0.66 (average 0.61) for non‐control wells, illustrating a high rate of reproducibility between replicates. Next, we identified synthetic lethal interactions by filtering the results for dsRNA reagents that reduced the viability of dNF1‐KO cells (median *Z* < −1.5) to a greater extent than wild‐type cells (median *Z* ≥ −1.5). This analysis identified 134 candidate genes (Table S1).

**Fig. 2:**
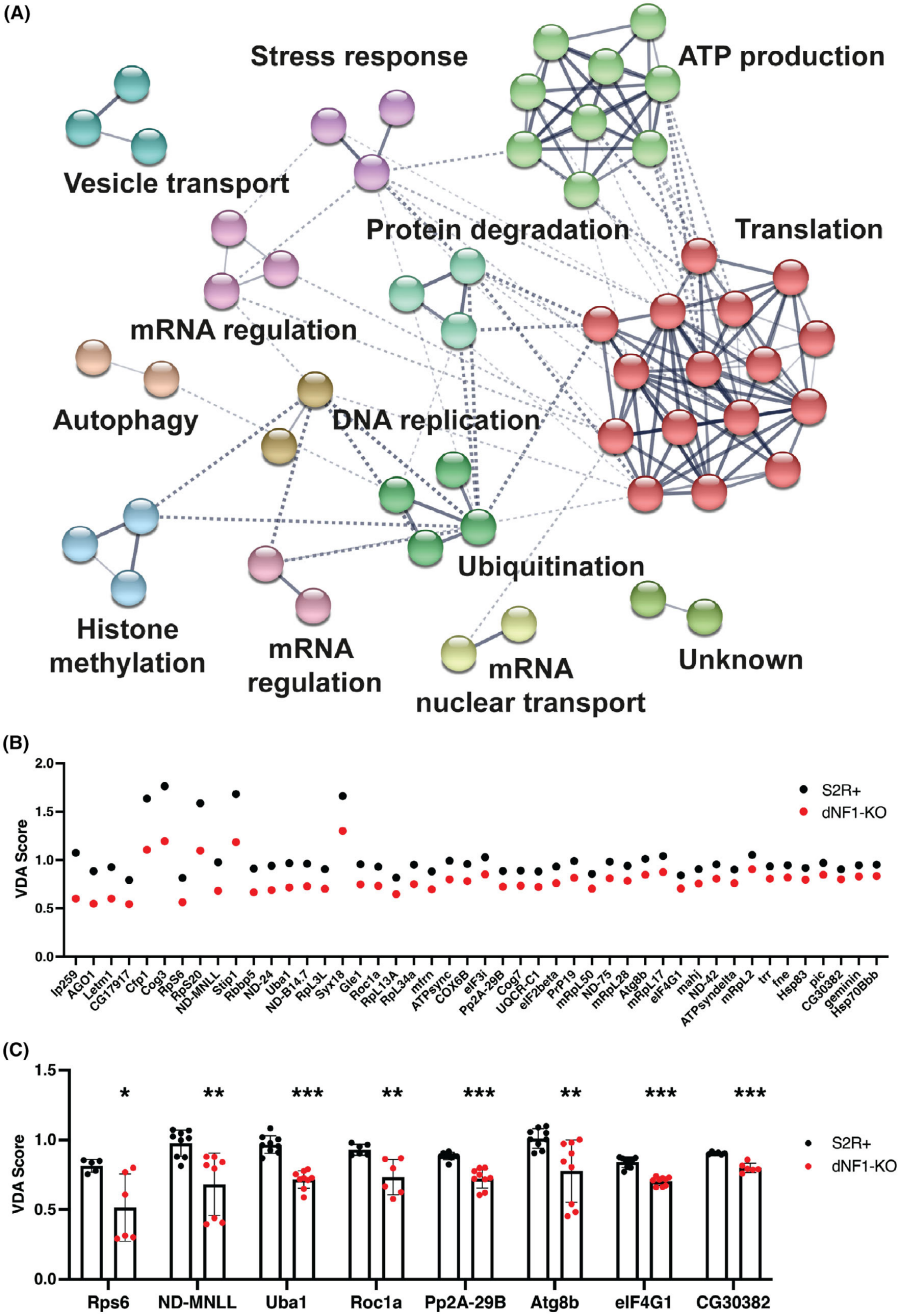
A network of synthetic lethal interaction for *Drosophila dNF1* and VDA analysis of candidate genes. Using wild‐type S2R+ and dNF1‐KO cells, we used a near genome‐wide dsRNA library to screen approximately 10 000 genes for a difference in viability phenotype in the absence of dNF1 compared to wild‐type cells. This screen identified 134 genes as having a synthetic lethal interaction with dNF1. (A) A synthetic lethal interaction network for dNF1 in *Drosophila* S2R+ cells generated using Cytoscape [101]. Solid lines represent physical interactions between proteins with similar functions and dashed lines represent physical interactions between proteins with unconnected functions. (B) Variable Dose Analysis (VDA) assays were performed for all 54 candidate genes, with two shRNAs per gene, in wild‐type (WT) S2R+ (black) and dNF1‐KO (red) cells. The best shRNA from the 46 genes that reduced dNF1‐KO viability by > 10% relative to S2R+ controls ranked in order of effect are shown. Data points indicate the average of nine replicates. (C) shRNA knockdowns of candidate genes that resulted in a > 10% reduction in dNF1‐KO viability compared to S2R+ controls and could be either directly targeted, or their associated pathways modulated, with existing drugs (number of replicates is indicated by the colored circles on each bar, error bars indicate standard deviation). All eight shRNAs showed a significant reduction in dNF1‐KO viability relative to S2R+ controls assessed using two‐tailed, unpaired *t*‐tests (*P < 0.05, **P < 0.01, ***P < 0.001).

Genetic screens are often associated with false‐positive results due to off‐target effects from dsRNA reagents or noise in the screen assay. To remove potential false positives, we used a similar approach to one used previously to assess synthetic lethal screen hits [24]. We overlaid the 134 screen hits onto a protein–protein interaction network from the STRING database [34]. Synthetic lethal interactions are generally similar between genes that have related functions; therefore, proteins that physically interact are expected to share synthetic lethal interactions. Using the combination of physical and genetic interaction data, we could remove false positives from the screen results by isolating only hits that have physical interactions with at least one other hit from the genetic interaction screen. In addition, we filtered the candidates to isolate only those with clear orthologs in humans. Following this process, 54 high‐confidence candidate targets remained, corresponding to 74 human genes (Fig. 2A, Table S2).

### 3.3 Validation of candidate synthetic lethal interactions using variable dose analysis (VDA)

We used the VDA assay as an additional combinatorial screen to assess synthetic lethality between *dNF1* and all 54 candidate drug targets. Two shRNAs targeting each of the genes were generated. These reagents were tested in S2R+ and dNF1‐KO cells. Of the 54 genes, 46 showed a > 10% reduction in viability in dNF1‐KO cells compared to S2R+ controls (Fig. 2B; ranked in order of effect on dNF1‐KO viability). These results indicate that the network is a reliable representation of the synthetic lethal interaction profile of the *dNF1* gene.

To identify potential drugs for repurposing to treat NF1 tumors, we filtered the candidate gene list to those associated with pathways that could be targeted using existing drugs, resulting in eight candidate genes (Fig. 2C). We then removed candidates that had previously been studied in relation to NF1, leaving five candidate genes. We also included MEK (selumetinib) as a control. Note that three drugs known to modulate the autophagy pathway were included, and some drugs inhibited multiple pathways. In total, we tested seven candidate drugs (Table 1).

**Table 1:** Candidate genes that selectively decreased dNF1‐KO viability without previous evidence of an interaction with NF1 that can be targeted with drugs, either directly or through pathway inhibition, with the exception of MEK (Selumetinib), which was included as a control.

Drug	Relevant synthetic lethal genes
	*Drosophila melanogaster* gene names are given with human orthologs in brackets
Chloroquine^a^	Autophagy; *Atg8b* (*GABARAP*), *RpS6* (*RPS6*)
EAD1	Autophagy; *Atg8b* (*GABARAP*), *RpS6* (*RPS6*)
Selumetinib^a^	*Dsor1* (*MEK*, *MAPKK*)
Metformin^a^	*ND‐MNLL* (*NDUFB1*)
PYR41	*Uba1* (*UBA1*)
LB100	*Pp2A‐29B* (*PPP2R1A* and *PPP2R1B*)
Bafilomycin A1	Autophagy; *Atg8b* (*GABARAP*), *RpS6* (*RPS6*)

^a^ FDA‐approved.

### 3.4 Existing autophagy drugs selectively affect *dNF1* ‐KO *Drosophila* cells

Repurposing existing drugs represents the most efficient route to develop new therapeutics. Each of the seven drugs that either directly inhibit the protein or the pathways associated with the *dNF1* synthetic lethal partner genes were first tested in S2R+ and dNF1‐KO cells using CellTiter‐Glo assays to measure viability after 48 h of treatment (Fig. S4A). Of the seven drugs tested in *Drosophila* cells, we selected CQ (an inhibitor of late‐stage autophagy) for further study as it showed a significant and consistent effect on reducing dNF1‐KO viability.

One of the strongest hits from the genetic screen was *Atg8b*, which encodes a key component of the autophagy pathway. Autophagy is commonly inhibited experimentally using CQ (or its derivative hydroxychloroquine), which is clinically used as an anti‐malarial and shows anti‐viral properties [35]. CQ functions to inhibit autophagy by blocking the binding of autophagosomes to lysosomes by diffusing into the lysosomes and altering the acidic environment, thereby inhibiting autophagic lysosomal degradation [36]. We initially focused on CQ because it is generally well‐tolerated [37] and can inhibit autophagy *in vivo* at clinically achievable concentrations [38].

**Fig. 3:**
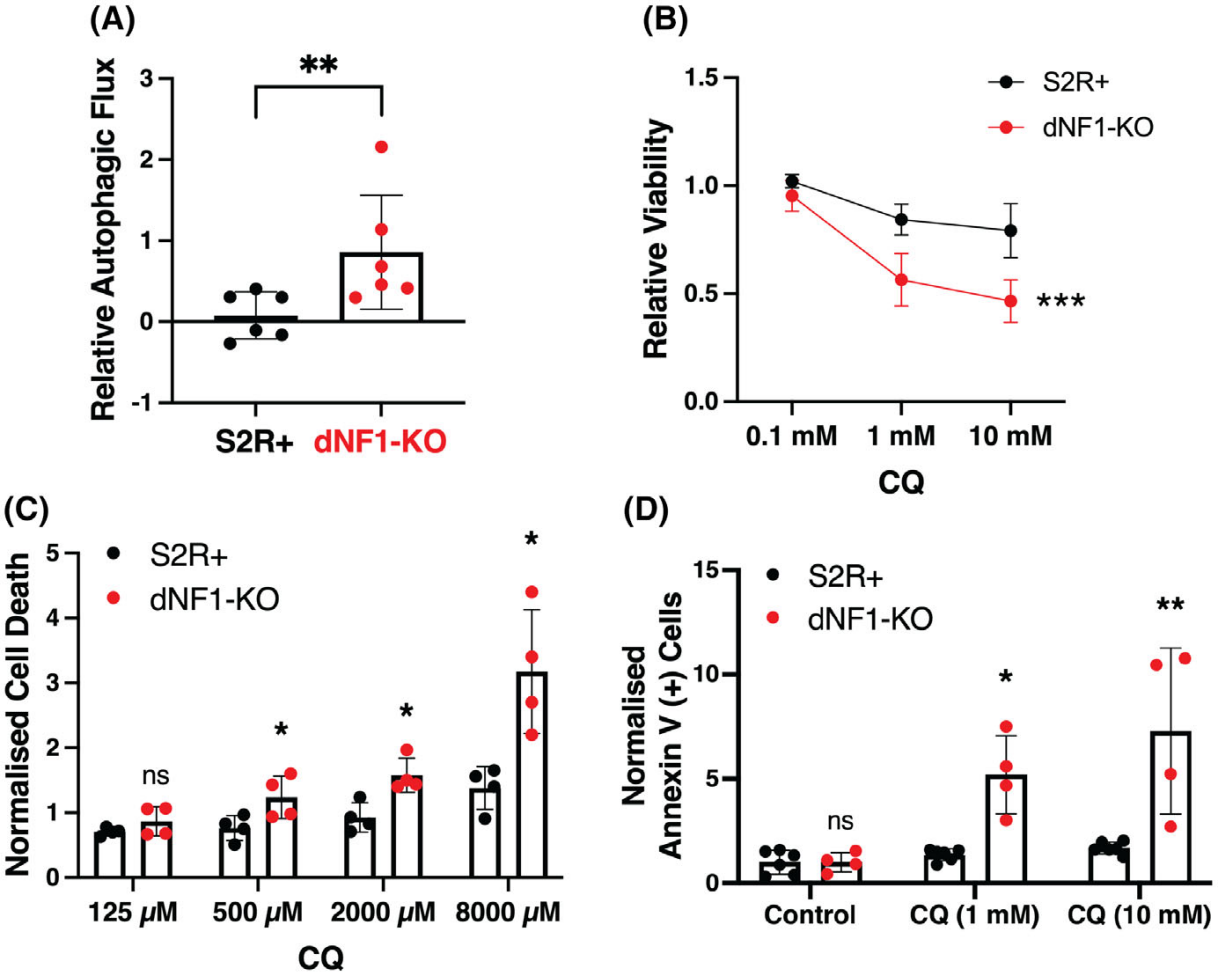
CQ selectively affects dNF1‐deficient *Drosophila* cells. (A) dNF1‐KO cells show a significant increase in autophagic flux compared to wild‐type (WT) S2R+ control cells in serum‐free media as assessed by inhibiting autophagosome flux for 4 h using the lysosomal inhibitor chloroquine (CQ) (10 μm), and then measuring the initial rate of accumulation of the fluorescent substrate labelling the autophagosomes using a fluorescent plate reader (normalized to DAPI, and then to S2R+ controls) (*n* = 6, **P < 0.01 obtained using the two‐tailed, unpaired, Student's *t*‐test). (B) CQ reduced dNF1‐KO cell viability compared to S2R+ cells after 48 h in serum‐free media as measured using CellTiter Glo assays (*n* = 4; ***P < 0.001 obtained using a two‐way ANOVA; the Tukey *post‐hoc* test for multiple comparisons showed *P* < 0.001 at both 1 and 10 mm CQ). (C) Analysis of cell death using propidium iodide (PI) staining with CQ in S2R+ and dNF1‐KO cells grown in serum‐free media (*n* = 4; *P < 0.05 obtained using the two‐tailed, unpaired, Student's *t*‐test comparing nNF1‐KO cells to S2R+ cells). (D) CQ increased annexin V staining in dNF1‐KO cells relative to S2R+ controls after 48 h in serum‐free media (*n* = 4 for control and 4 for CQ; *P < 0.05 and **P < 0.01 obtained using the two‐tailed, unpaired, Student's *t*‐test comparing dNF1‐KO cells to S2R+ cells). In all cases, bars represent the mean and error bars indicate standard deviation.

First, we quantified autophagic flux, i.e., how many autophagosomes form and then become degraded, by measuring the difference in the number of autophagic vesicles in the presence versus the absence of lysosomal inhibition using a high dose of CQ. We observed significantly higher levels of autophagic flux in dNF1‐KO cells under serum‐free conditions compared to S2R+ controls (Fig. 3A). We then tested whether CQ would phenocopy the selective effect observed using genetic inhibition of *Atg8b* in *Drosophila* cells. Both S2R+ and dNF1‐KO cells were treated with varying doses of CQ, which were chosen based on dose curves performed in pilot experiments (Fig. S4A) and cell viability was measured using CellTiter‐Glo assays, PI staining, and annexin V staining. A significantly greater effect on dNF1‐KO cell viability was observed at multiple concentrations of CQ in serum‐free media after 48 h of treatment across all three assays, further validating the interaction between autophagy and NF1 and demonstrating that the effect is cytotoxic (Fig. 3B–D).

### 3.5 Both early‐ and late‐stage autophagy genes have a synthetic lethal interaction with *dNF1* , which can be targeted with other autophagy inhibitors

**Fig. 4:**
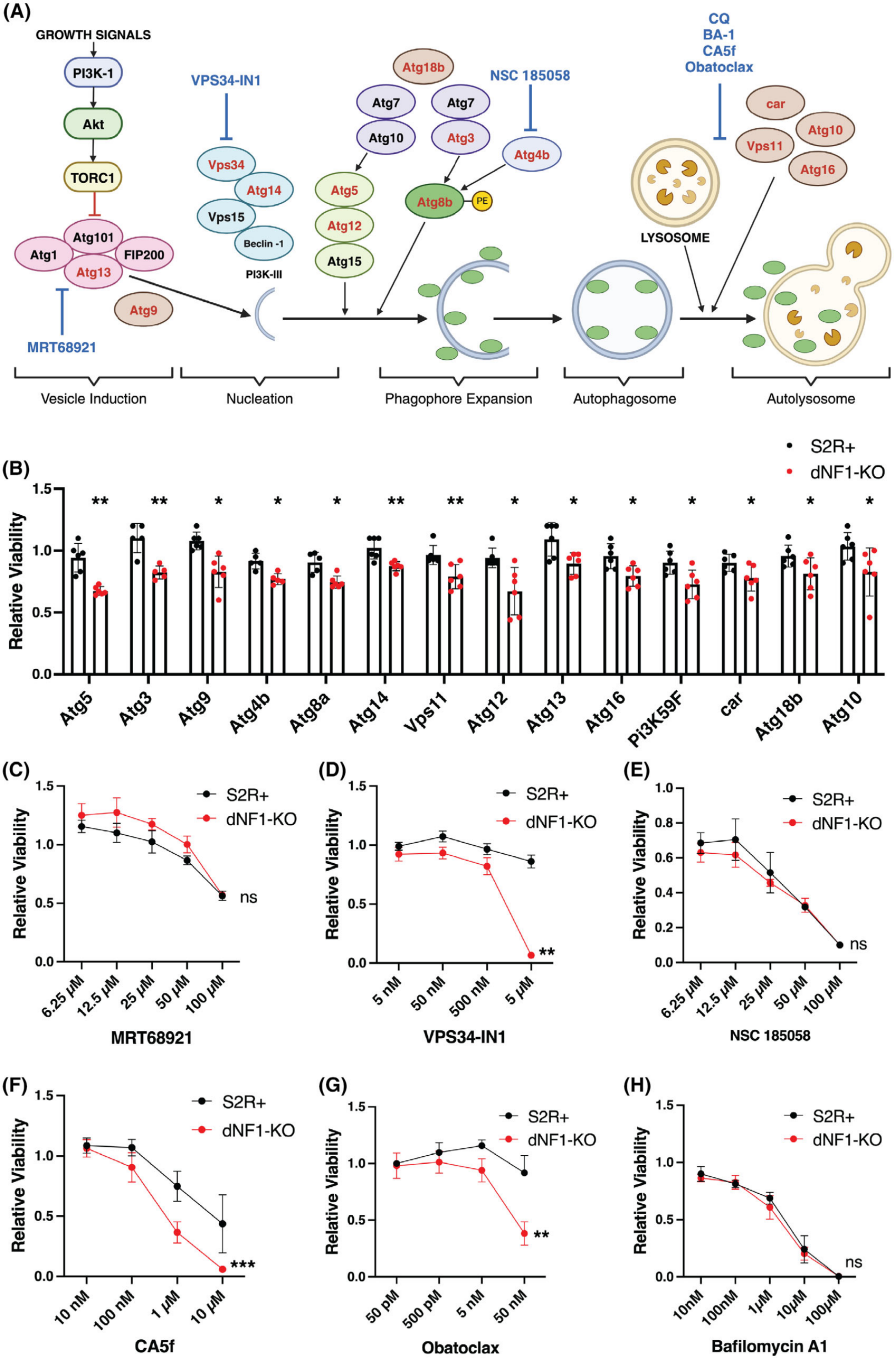
Both early‐ and late‐stage autophagy genes have a synthetic lethal interaction with *dNF1*, which can be targeted with other drugs known to inhibit autophagy. (A) Diagrammatic representation of the autophagy pathway in *Drosophila*. The genes found to have a synthetic lethal interaction with *dNF1* are shown in red. Drugs used to target each stage of the pathway in subsequent viability assays are shown in blue. (B) *Variable Dose Analysis* (VDA) assays performed for 30 autophagy‐related genes, with two shRNAs per gene, in S2R+ (black) and dNF1‐KO (red) cells. Shown is the most effective shRNA from each of the 14 genes that reduced dNF1‐KO viability by > 10% relative to S2R+ controls, ranked in order of effect. Bars indicate the average of 6 replicates and error bars indicate standard deviation. *P*‐values were calculated using two‐tailed, unpaired *t*‐tests; *P < 0.05, **P < 0.01. (C–G) Testing drugs that target the autophagy pathway for differences in cell viability between dNF1‐KO and S2R+ cells after 48 h in serum‐free media as measured using the CellTiter Glo assays (*n* = 4; **P < 0.01, ***P < 0.001 obtained using a two‐way ANOVA). VPS34‐IN1 (D), CA5f (F), and obatoclax (G) selectively reduced dNF1‐KO cell viability compared to S2R+ cells in a dose‐dependent manner. By contrast, MRT68921 (C), NSC 185058 (E), and bafilomycin A1 (H) did not selectively affect dNF1‐KO cell viability compared to S2R+ cells. In all cases, data represents the mean and error bars indicate standard deviation.

Autophagy is a complex process involving multiple stages and many different proteins (Fig. 4A). To confirm that disruption of autophagy causes a selective effect on *dNF1*‐KO cells and to determine whether more specific targeting of autophagy components would result in a greater selective effect, we performed an additional VDA screen to assess for synthetic lethal interactions between *dNF1* and 29 key autophagy genes (in addition to *Atg8b*) using S2R+ and dNF1‐KO cells (all 29 genes screened are shown in Table S3). In total, knockdown of 14 genes, in addition to the previously identified *Atg8b*, were found to significantly reduce dNF1‐KO viability by > 10% relative to controls (Fig. 4B). Interestingly, these 14 genes are implicated across multiple stages of the autophagy pathway (Fig. 4A, genes shown in red). Therefore, we tested drugs known to modulate specific aspects of the autophagy pathway in dNF1‐KO cells. These included (a) MRT68921 (a specific and potent inhibitor of ULK1 that prevents formation of the ULK1 complex in the early‐stages of autophagy [39]), (b) VPS34‐IN1 (a highly selective inhibitor of VPS34, preventing the formation of the PI3K‐III complex [40]), (c) NSC 185058 (a reported inhibitor of ATG4B; however, although reported to inhibit the lipidation of LC3B, resulting in the inhibition of autophagy via a mechanism that is independent of mTOR [41], one study also reported that it failed to inhibit ATG4B directly [42]), (d) CA5f (a late‐stage inhibitor of autophagic flux shown to be effective *in vitro* and *in vivo* [43]), (e) obatoclax (a pan‐Bcl‐2 inhibitor reported to indirectly yet potently inhibit late‐stage autophagy [44]), and (f) bafilomycin A1 (disrupts autophagic flux by independently inhibiting V‐ATPase‐dependent acidification and Ca‐P60A/SERCA‐dependent autophagosome‐lysosome fusion [45]) (Fig. 4A). The dose ranges were selected based on the published IC_50_ values for each drug. In general, the above‐mentioned drugs showed a selective lethal effect in dNF1‐KO cells, except for MRT68921, NSC 185058, and bafilomycin A1 (Fig. 4C–H); however, none of the drugs were deemed to be more effective in selectively killing dNF1‐KO cells than CQ. CQ also has the advantage of being FDA‐approved. Therefore, although targeting autophagy at various stages of the pathway appears to have selective lethality effects in dNF1‐KO cells, CQ showed the greatest potential for use in the clinic to treat NF1.

### 3.6 CQ and bafilomycin A1 selectively affect *NF1* ‐deficient human cells

To determine whether the selective effect of CQ was conserved in human cells, we tested the effects of drug treatment on a panel of human *NF1*‐deficient cell lines. These included a pair of otherwise isogenic *NF1* ^*+/−*^ (C8) and *NF1* ^*−/−*^ (C23) immortalized Schwann cells generated using CRISPR/Cas9 gene editing (Fig. 1E–G, Fig. S3C,D). In addition, we used two NF1 patient‐matched pairs of immortalized Schwann cell lines derived from PNs (pair 1: ipnNF95.11C (*NF1* ^*+/−*^) and ipNF95.11b ‘C’ (*NF1* ^*−/−*^) and pair 2: ipnNF09.4 (*NF1* ^*+/−*^) and ipNF05.5 (*NF1* ^*−/−*^)) [21]. Doses were selected based on pilot assays assessing the effects of the drugs over a range of concentrations (Fig. S4B,C). Similar to CQ, bafilomycin A1 is a potent inhibitor of autophagosome‐lysosome fusion but is not clinically approved [46]. Although bafilomycin A1 showed no significant effects in *Drosophila* dNF1‐KO cells, a selective effect was observed in the panel of human *NF1*‐deficient cell lines (Fig. S4A–C); therefore, we also tested bafilomycin A1 to provide additional validation of the effects on autophagy through autophagosome‐lysosome fusion inhibition brought about through an independent mechanism.

**Fig. 5:**
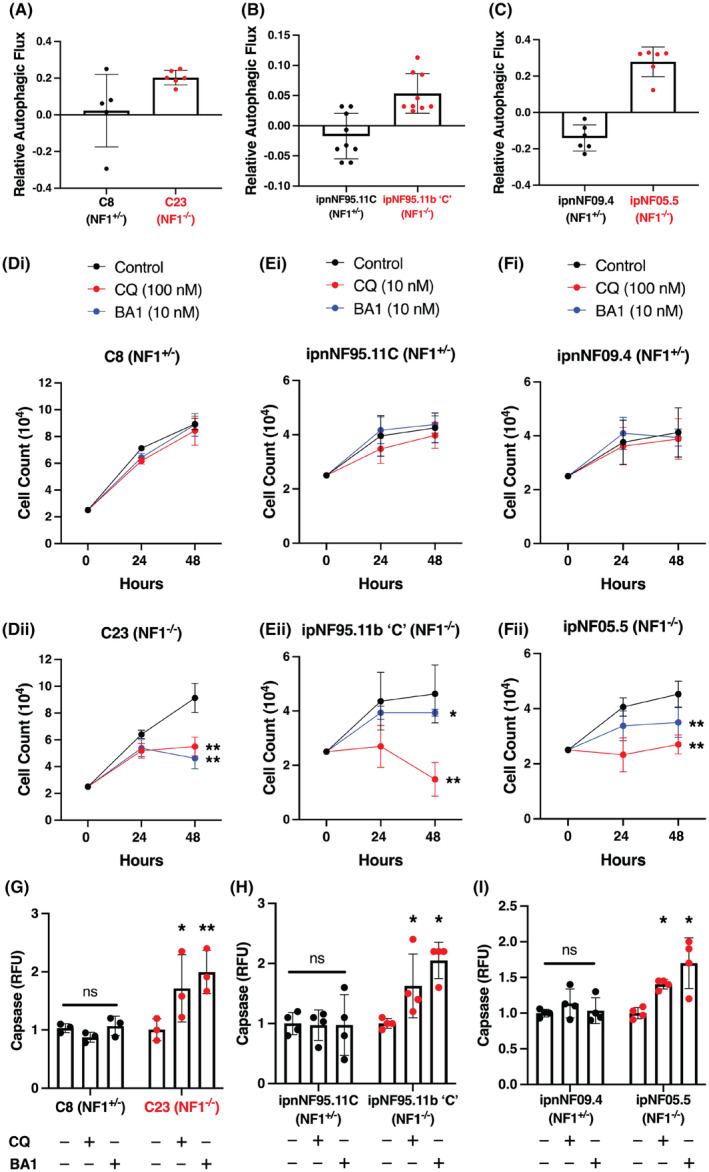
CQ and bafilomycin A1 (BA1) selectively affect *NF1*‐deficient human cells. (A–C) Autophagic flux was increased in C23, ipNF95.11b ‘C’, and ipNF05.5 NF1^−/−^ cells compared to heterozygous controls after 4 h of lysosomal inhibition with chloroquine (CQ) (10 μm) in serum‐free media (number of replicates is indicated by the colored circles on each bar; ***P < 0.001 obtained using the two‐tailed, unpaired, Student's *t*‐test; ns = non‐significant). (Di‐Fii) Cell counts over 48 h revealed that both CQ and Bafilomycin‐A1 (BA1) significantly reduced NF1^−/−^ cell viability relative to NF1^+/−^ controls at varying doses in C8 and C23 cells (*n* = 4) (Di‐Dii), ipnNF95.11C/ipNF95.11b ‘C’ cells (*n* = 8 for control, 5 for CQ and 4 for BA1) (Ei‐Eii), and ipnNF09.4/ipNF05.5 cells (*n* = 8 for control, 4 for CQ and 4 for BA1) (Fi‐Fii); *P < 0.05, **P < 0.01 obtained using a two‐way ANOVA with the Tukey *post‐hoc* test for multiple comparisons). (G–I) We observed an increase in caspase activation in NF1^−/−^ cells relative to NF1^+/−^ control cells when treated with CQ or BA1 for 48 h in serum‐free media (replicates are indicated by the colored circles on each bar). (G), C8 and C23 cells (50 and 5 nm, respectively), (H) ipnNF95.11C/ipNF95.11b ‘C’ cells (1 and 5 nm, respectively), and (I) ipnNF09.4/ipNF05.5 cells (50 and 5 nm, respectively) (*n* = 4; *P < 0.05, **P < 0.01 obtained using a one‐way ANOVA with the Tukey *post‐hoc* test for multiple comparisons). In all cases, bars represent the mean and error bars indicate standard deviation.

Autophagic flux was significantly higher in *NF1* ^*−/−*^ cells (ipNF95.11b ‘C’ and ipNF05.5) relative to *NF1* ^*+/−*^ controls (ipnNF95.11C and ipnNF09.4), with a similar but not significant effect also observed in the CRISPR‐generated *NF1* ^*−/−*^ (C23) cells compared to *NF1* ^*+/−*^ (C8) controls (Fig. 5A–C). Similarly, lysosomal activation was increased in ipNF95.11b ‘C’ cells compared to ipnNF95.11C controls, further indicating an increase in baseline autophagy levels, which was inhibited by CQ and bafilomycin A1 in both *NF1* ^*+/−*^ and *NF1* ^*−/−*^ cells (Fig. S5A). To further verify the dysregulation of autophagic flux in NF1‐deficient cells, we assessed the baseline expression of LC3B, a structural protein of autophagosomal membranes, widely used as biomarker of autophagy, with western blotting, which was found to be increased in *NF1* ^*−/−*^ cells (ipNF95.11b ‘C’) relative to *NF1* ^*+/−*^ control (ipnNF95.11C) (Fig. S5B).

Both CQ and bafilomycin A1 resulted in a significantly greater reduction in the viability of *NF1* ^*−/−*^ deficient cells compared to *NF1* ^*+/−*^ controls and WT Schwann cells after 24 and 48 h of treatment under serum‐free media conditions, as measured with cell counts and caspase assays (Fig. 5D–I, Fig. S4D), with the most pronounced effect observed at 48 h. We observed some variation in the most effective dose of CQ between cell lines, with the ipNF95.11b ‘C’ cells showing a selective effect at a 10‐fold lower dose compared to the other two human cell lines. Collectively, this demonstrates that the selective effects are conserved between *Drosophila* and human systems.

We also tested the additional five drugs that target the autophagy pathway in the two patient *NF1*‐deficient cell lines (Fig. S6). While all of these drugs showed selective lethal effects in at least one model cell line, no drug showed a consistent effect across the panel of human cell lines that was comparable to that of CQ and bafilomycin A1, further highlighting the reproducibility of our *Drosophila* model system.

Together, these results demonstrate that *NF1*‐deficient cells have a vulnerability to disruption of the autophagy pathway, which is conserved and reproducible with multiple drugs that modulate autophagy between *Drosophila* and human Schwann cells derived from NF1‐associated tumors. Not only does autophagy represent a promising pathway for targeting *NF1*‐deficient tumors, but also we identified CQ as a candidate drug for the potential treatment of NF1 tumors.

### 3.7 CQ causes selective viability effects at lower doses than selumetinib

**Fig. 6:**
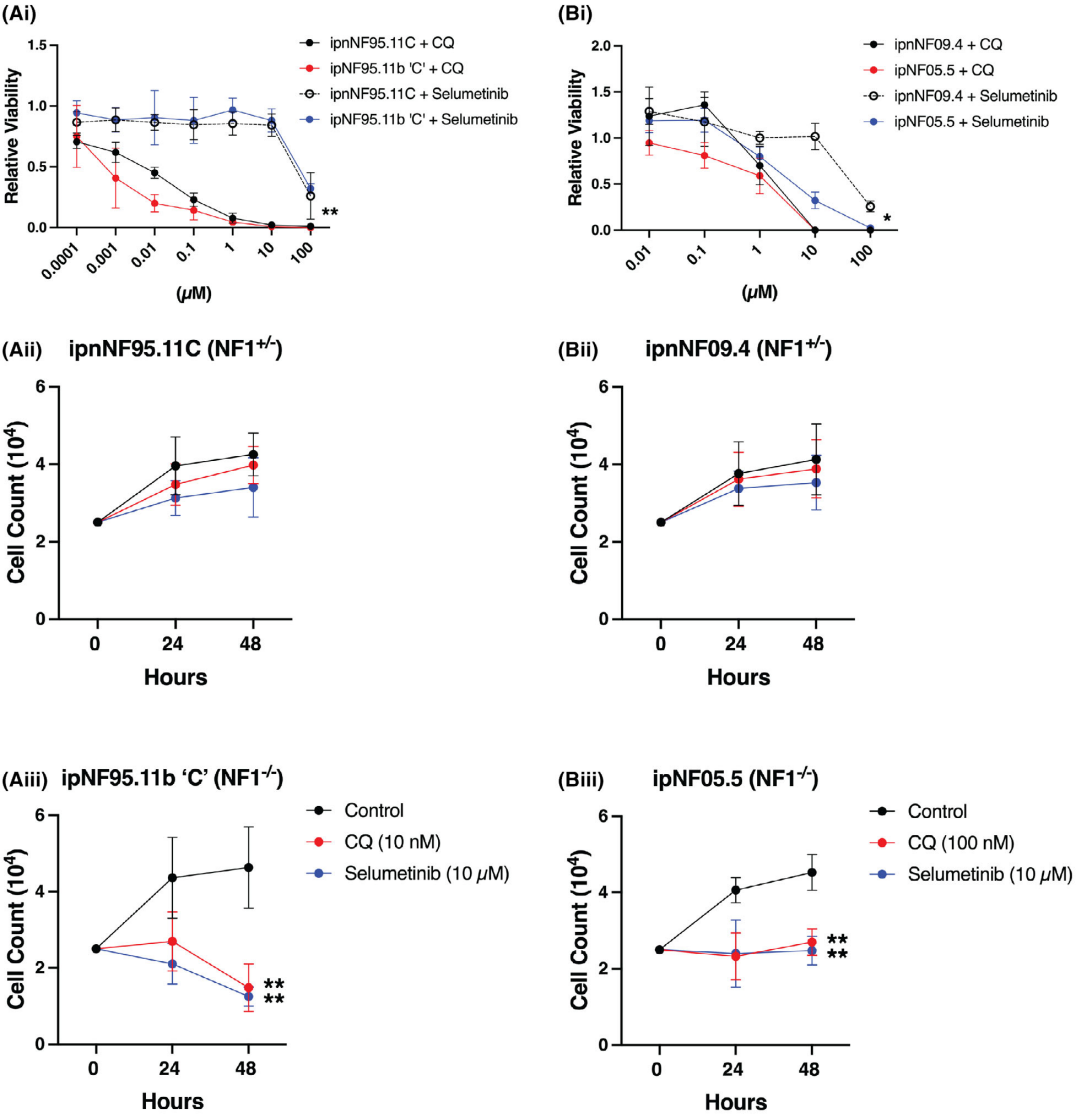
CQ reduces NF1‐deficient cell proliferation to a greater extent than selumetinib. (Ai and Bi) CellTiter Glo viability results between two human NF1‐deficient cell lines and their heterozygous controls treated for 48 h with chloroquine (CQ) or selumetinib in serum free media are compared. Concentration of the drugs is shown on the *x*‐axis and data are normalized to the no‐drug controls (*n* = 8; *P < 0.05, **P < 0.01 obtained using a two‐way ANOVA). (Aii–Biii) In a similar experiment, cell numbers were assessed in response to treatment with CQ (10/100 nm) or selumetinib (10 μm) at 24 and 48 h. Both drugs effectively prevented the proliferation of NF1‐deficient cells (*n* = 8 for control, 6 for CQ and 4 for selumetinib; **P < 0.01 obtained using a two‐way ANOVA with the Tukey post‐test for multiple comparisons). In all cases, error bars represent the standard deviation.

To determine how CQ compares to the only drug currently approved by the FDA for the treatment of NF1 tumors, selumetinib, we compared the effects of each drug on *NF1*‐deficient cell viability using three human cell lines, each with a paired isogenic or patient‐matched control. In all three *NF1‐*deficient cell lines (C23, ipNF95.11b ‘C’, and ipNF05.5), CQ resulted in selective lethal effects at approximately 100–1000‐fold lower concentration (1–100 nm) than selumetinib (10–100 μm) after 48 h treatment in serum free media, measured using CellTiter Glo assays (Fig. 6A,B, Fig. S4E). The relatively weak effects of selumetinib in these cells is consistent with previous reports using ipNF95.11b ‘C’ and ipNF05.5 cells [47, 48]. Furthermore, the effect of CQ was found to be more consistently selective, i.e., a significant difference was observed between all three *NF1* ^*+/−*^ and *NF1* ^*−/−*^ cell pairings treated with CQ, whereas selumetinib only resulted in a significant difference in ipNF05.5 cells vs. ipnNF09.4 controls, and not in the other two cell lines. Finally, in two of the cell line pairs (C8/C23 and ipnNF95.11C/ipNF95.11b ‘C’), CQ treatment resulted in a greater difference in viability between the NF1‐deficient and control cell line, indicating a more potent effect. The strength of the selective effect in the third cell line pair (ipnNF09.4/ipNF05.5) was comparable between the two drugs.

To further compare the drugs, we performed live cell counts of ipnNF95.11C (*NF1* ^*+/−*^) and ipNF95.11b ‘C’ (*NF1* ^*−/−*^) treated with CQ (10 nm) and selumetinib (10 μm), and ipnNF09.4 (*NF1* ^*+/−*^) and ipNF05.5 (*NF1* ^*−/−*^) cells treated with CQ (100 nm) and selumetinib (10 μm), at 24 and 48 h. Upon treatment with each drug, both *NF1* ^*+/−*^ cell lines continued to proliferate over 48 h (Fig. 6Aii,Bii). However, the number of ipNF95.11b ‘C’ (*NF1* ^*−/−*^) cells decreased and ipNF05.5 (*NF1* ^*−/−*^) cells remained unchanged over 24 and 48 h, with comparable effects observed between the two drugs (Fig. 6Aiii,Biii). In summary, CQ shows evidence of increased potency and more consistent effects between cell models at lower concentrations compared to selumetinib when tested using CellTiter Glo assays, and the effects on cell numbers are comparable. Therefore, CQ is a promising candidate with potential for further development towards clinical use for the treatment of NF1 tumors.

### 3.8 CQ affects survival in a *Drosophila in vivo NF1* mutant model

**Fig. 7:**
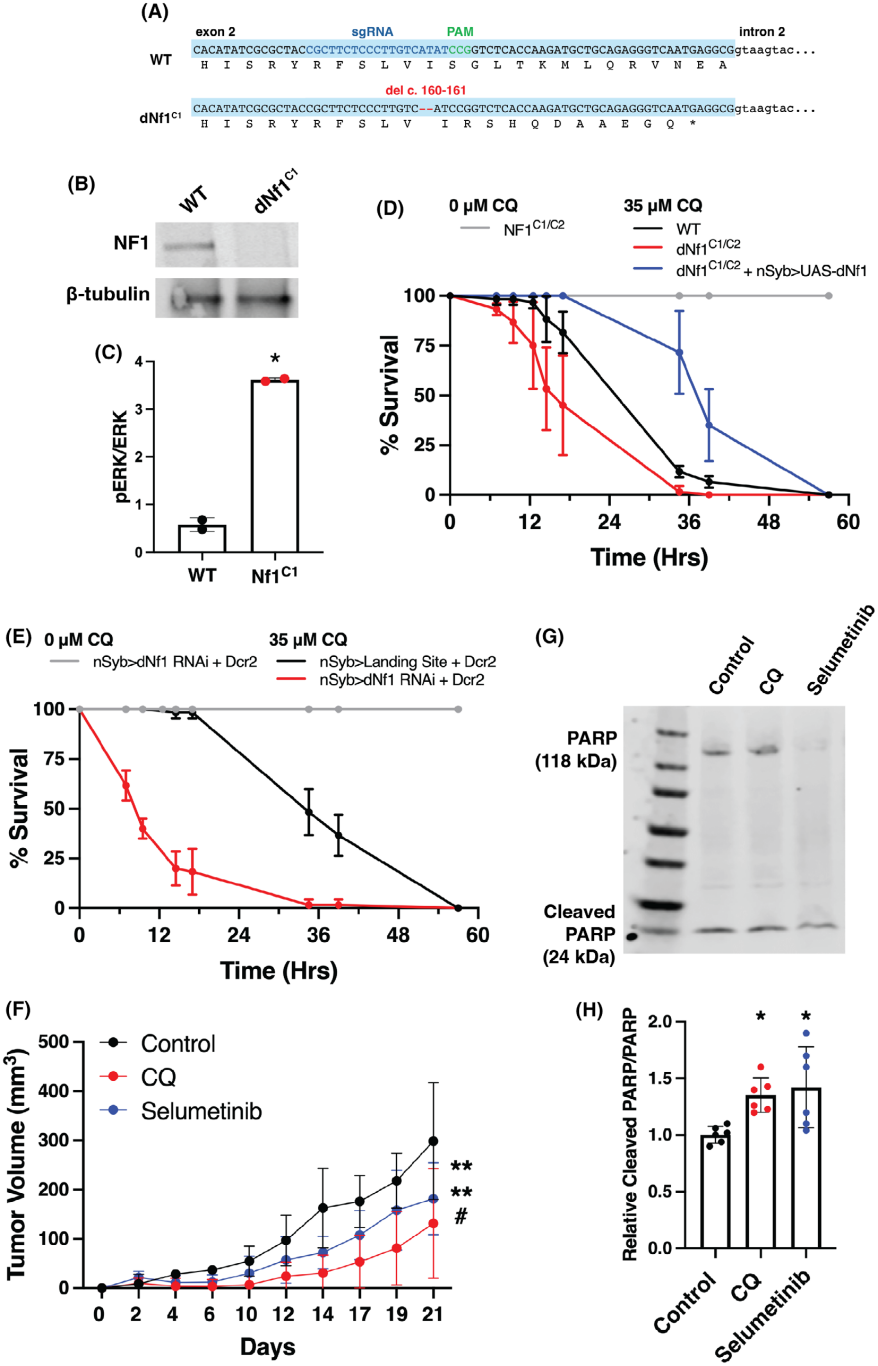
CQ affected lethality in NF1 mutant *Drosophila* and reduced *NF1*‐deficient MPNST tumor xenograft growth *in vivo*. (A) A novel dNf1 null mutant was generated using CRISPR gene editing: dNf1^C1^ (dNF1 delAT162‐163). A double‐stranded break was targeted to exon 2 of *dNF1*. Molecular analysis of the resulting dNf1^C1^ line revealed a 2 bp deletion, resulting in a frame shift and premature termination within exon 2. The position of the guide RNA is shown in blue and the proximal adjacent motif (PAM) site in green. (B) Western blots of anti‐dNf1 immunoprecipitates from lysates prepared from adult heads from CRISPR mutants showed no expression of Nf1 in *dNF1* ^*C1*^ homozygous animals. (C) ELISA for pERK/ERK showed a fourfold increase in the pERK/ERK ratio in adult heads from dNf1^C1^ mutants compared to wild‐type (WT) flies. Error bars indicate the standard deviation between duplicate samples. A paired, two‐tailed *t*‐test was performed to determine significance; *P < 0.05. (D) Addition of 35 μm chloroquine (CQ) to food resulted in increased lethality of dNf1^C1^ mutants compared to WT flies (*n* = 3, 20 flies per replicate). The sensitivity of dNf1 mutant flies to CQ was rescued by re‐expression of dNf1 from a UAS‐dNF1 transgene using the nSyb‐Gal4 driver (*n* = 3, 20 flies per replicate). Error bars indicate standard deviation. (E) dNf1 RNAi knockdown flies (nSyb‐Gal4 > v109637) show a similarly reduced survival time when cultured on food with 35 μm CQ compared to control flies (nSyb‐Gal4 > VIE‐260B v60100). *n* = 3, 20 flies per replicate. Error bars indicate standard deviation. (F) In mice implanted with ST88‐14 NF1^−/−^ cells, intraperitoneal injections of CQ (50 mg·kg^−1^, 3× weekly) or oral gavage with selumetinib (25 mg·kg^−1^, 3× weekly) significantly slowed tumor growth compared to vehicle‐treated controls. Furthermore, there was a significant reduction in tumor growth in CQ‐treated mice compared to selumetinib‐treated mice (*n* = 6; **P < 0.01 vs control, and ^#^ *P* < 0.05 CQ vs selumetinib, obtained using a two‐way ANOVA). Error bars indicate standard deviation (G, H) Following extraction of the xenografts, western blotting revealed the increased protein expression of the 24 kDa fragment of cleaved PARP relative to PARP in CQ and selumetinib‐treated mice in comparison to controls (*n* = 6; *P < 0.05 obtained using the one‐way ANOVA). Error bars indicate standard deviation (H).

As CQ is FDA‐approved, well tolerated, and showed a significant effect on dNF1‐KO and human NF1^−/−^ cell viability, we chose to take this drug forward to determine synthetic lethality *in vivo*. *Drosophila* mutant flies show defective Ras signaling, which result in several neurobehavioral phenotypes [49, 50, 51, 52, 53]. For this study, we generated a novel *dNf1* null mutant fly line using CRISPR gene editing: *dNf1* ^*C1*^ (delAT162‐163) (Fig. 7A). Western blots using lysates prepared from adult heads from *dNf1* ^*C1*^ homozygous mutants showed no expression of neurofibromin (Fig. 7B). In addition, ELISAs showed a fourfold increase in pERK/ERK of *dNf1* ^*C1*^ mutants compared to the WT parental line (Fig. 7C).

To determine whether CQ affects survival in *dNF1*‐deficient *Drosophila*, we took two approaches. Firstly, we compared the effect of CQ (35 mm) on *dNf1* ^*C1*^ homozygous null mutant flies, the WT parental line, and *dNf1* ^*C1*^ with re‐expression of dNf1 from a *UAS‐dNF1* transgene driven with a pan‐neuronal (*nSyb‐Gal4*) driver (*dNf1* ^*C1*^ + *nSyb‐Gal4 > UAS‐dNf1*). CQ resulted in increased lethality of *dNf1* ^*C1*^ mutants compared to the WT control in a dose‐dependent manner (Fig. 7D and Fig. S7). Furthermore, we were able to rescue the CQ sensitivity of *dNf1* ^*C1*^ mutant flies by re‐expression of dNf1 from a *UAS‐dNF1* transgene (Fig. 7D). Secondly, we tested flies with pan‐neuronal RNAi knock down of *dNf1* (using *nSyb‐Gal4*) compared to a landing site control on food containing CQ (35 mm). Flies with dNf1 RNAi knockdown, showed significantly reduced survival time on CQ compared to CQ‐treated landing site control flies, and untreated flies, phenocopying the effects seen in *dNf1* ^*C1*^ mutant flies (Fig. 7E). Together, these results demonstrate that *dNf1*‐deficient flies have vulnerability to CQ treatment, as shown to be conserved and reproducible in *Drosophila* NF1‐KO cells and human Schwann cells derived from NF1‐associated tumors. This further highlights the autophagy pathway as a target for the potential treatment of NF1‐associated tumors, with CQ as a candidate drug.

### 3.9 CQ reduced *NF1* ‐deficient MPNST tumor xenograft growth *in vivo*

In order to test the effects of CQ *in vivo* in a mammalian model, we used the ST88‐14 *NF1* ^*−/−*^ MPNST xenograft mouse model. Although some NF1 PN tumor cells have been shown to form xenograft tumors, they are very slow growing and require signaling from *NF1* ^*−/−*^ peripheral nerves within the tumor microenvironment [54]. On the other hand, highly aggressive MPNST *NF1* ^*−/−*^ cells grow rapidly in matrigel xenografts, resulting in a highly reproducible preclinical model that allows the continuous quantitation of tumor growth during the study period; therefore, we chose to use this model to assess the effects on CQ on tumor growth. In mice implanted with ST88‐14 *NF1* ^*−/−*^ cells, treatment with CQ (50 mg·kg^−1^ in saline, intraperitoneally, three times per week) or selumetinib (25 mg·kg^−1^ in saline, oral gavage, three times per week) once the tumors had started to grow, resulted in a significant reduction in tumor cell growth over a period of 3 weeks in comparison to vehicle‐treated controls (Fig. 7F). In addition, both CQ and selumetinib treatments induced a marker of cell death in the tumors (Fig. 7G,H). Furthermore, there was a significant reduction in tumor cell growth in CQ‐treated mice in comparison to selumetinib‐treated mice, indicating CQ to have a superior effect on *NF1*‐mutant tumor growth. CQ and selumetinib treatment were found to have no toxicity effects in these mice (Fig. S8), which was also assessed in a prior study on C57BL/6 mice (data not shown).

CQ and selumetinib were found to induce *NF1*‐deficient cell apoptosis *in vitro*; therefore, we used western blotting to assess the cleavage of a key apoptosis protein, PARP, in xenografts from control, CQ, and selumetinib‐treated mice. We found a significant increase in the 24 kDa fragment of cleaved PARP relative to total PARP in xenografts from both CQ and selumetinib‐treated mice, indicating that both drugs resulted in cell apoptosis in the NF1 xenografts, thus slowing tumor growth.

### 3.10 Selumetinib enhances the viability effect of CQ and bafilomycin A1

**Fig. 8:**
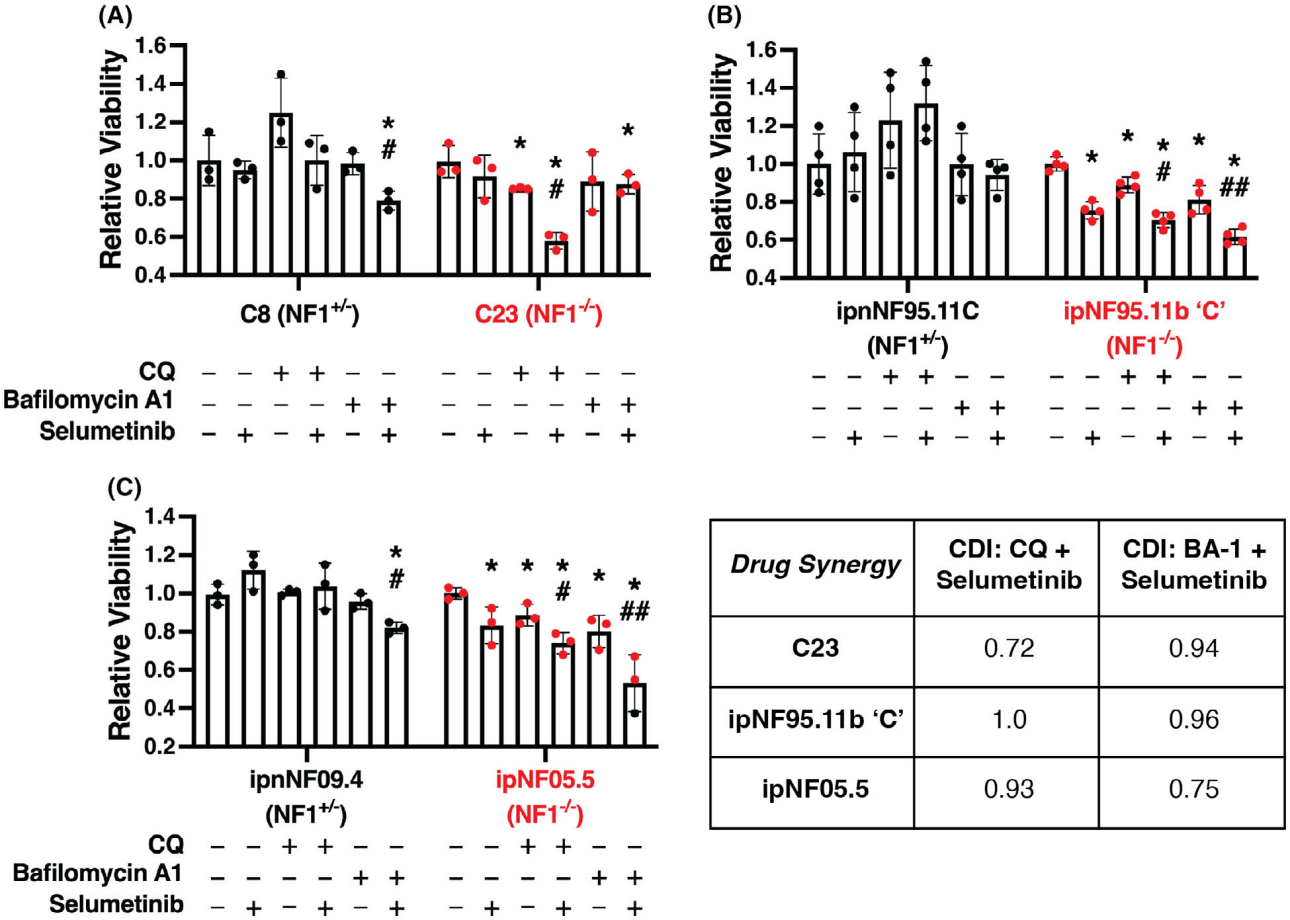
Combined effect of selumetinib with CQ or bafilomycin A1 on *NF1*‐deficient cell viability. (A) Control (NF1^+/−^) and NF1^−/−^ cells were treated with selumetinib combined with chloroquine (CQ) or bafilomycin A1 for 48 h in serum‐free media. Selumetinib alone had no effect on the viability of C8, C23 (10 μm), ipnNF95.11C (5 μm), and ipnNF09.4 (10 μm) cells (A–C); however, it did significantly decrease viability of ipNF95.11b ‘C’ and ipNF05.5 NF1^−/−^ (5 μm) cells, as measured using CellTiter Glo assays. (B, C). The effect of CQ was enhanced when combined with selumetinib in all three NF1^−/−^ cell lines (50 nm, 100 pm, and 50 nm, respectively), whereas the effect of bafilomycin A1 (all 1 nm, respectively) was enhanced by selumetinib only in the patient‐derived NF1‐deficient cell lines (ipNF95.11b ‘C’ and ipNF05.5) (*P < 0.05 and **P < 0.01 vs control, ^#^ *P* < 0.05 and ^##^ *P* < 0.01 vs single drug (CQ or bafilomycin A1), obtained using a two‐way ANOVA with Tukey for multiple comparisons). In all cases, bars represent the mean (*n* = 3 (A,C) and 4 (B)) and error bars indicate standard deviation. (D) The coefficient of drug interaction (CDI) between CQ + selumetinib or bafilomycin A1 + selumetinib was calculated to determine whether drug synergy was present. CDI < 1 indicates synergy (with CDI < 0.7 indicating a strong synergistic effect), CDI = 1 indicates additivity, and CDI > 1 indicates antagonism. In this case, drug pairs were classed as synergistic if CDI < 0.95. CQ and selumetinib showed some degree of synergy in C23 cells, whereas an additive effect was observed in ipNF95.11b ‘C’ and ipNF05.5 cells. Bafilomycin A1 and selumetinib showed some degree of synergy in ipNF05.5 cells, and an additive effect in ipNF95.11b ‘C’ and C23 cells.

Selumetinib, a MEK1/2 inhibitor, is currently the only FDA‐approved drug for the treatment of tumors associated with NF1 [55]. We treated *NF1* ^*+/−*^ and *NF1* ^*−/−*^ cells with selumetinib with and without CQ or bafilomycin A1 for 48 h in serum‐free media and performed CellTiter‐Glo assays to measure cell viability (Fig. 8A–C). In C8/C23, ipnNF9511.C/ipNF95.11b ‘C’, and ipnNF09.4/ipNF05.5 cell lines, selumetinib significantly enhanced the reduced viability effect of CQ in *NF1* ^*−/−*^, but not *NF1* ^*+/−*^ cells (Fig. 8A–C). Therefore, combined treatment of CQ and selumetinib had a greater impact in *NF1*‐deficient cell viability compared to either drug alone. In addition, selumetinib significantly enhanced the viability effect of bafilomycin A1 in patient *NF1* ^*−/−*^ (ipNF95.11b ‘C’ and ipNF05.5) but not *NF1* ^*+/−*^ cells (ipnNF9511.C and ipnNF09.4), although not in C8/C23 cells (Fig. 8A–C).

To determine whether these combined effects were additive or synergistic, the coefficient of drug interaction (CDI) was calculated for each cell line treated with CQ + selumetinib or bafilomycin A1 + selumetinib. CDI was calculated as follows: CDI = AB/(A × B) [56, 57, 58], where A is fold change of CQ or bafilomycin A1 compared to no‐drug control, B is fold change of selumetinib compared to no‐drug control and AB is the fold change of the combined treatment compared to no‐drug control. A CDI < 1 indicates synergy, CDI = 1 indicates additivity, and CDI > 1 indicates antagonism. CQ and selumetinib showed some degree of synergy in C23 cells, whereas an additive effect was observed in ipNF95.11b ‘C’ and ipNF05.5 cells. Bafilomycin and selumetinib showed some degree of synergy in ipNF05.5 cells, and an additive effect in ipNF95.11b ‘C’ and C23 cells (Fig. 8D).

## 4 Discussion

In this study, we used a novel *Drosophila* cell culture model of NF1 to identify new potential therapeutic targets and subsequently candidate drugs for the selective killing of *NF1*‐deficient cells. Using genetic screening of *Drosophila* cells, we identified 54 candidate synthetic lethal genes that when knocked down resulted in the death of *NF1‐*deficient cells without impacting the viability of wild‐type, healthy cells. Of these genes, 85% (46/54) were validated in secondary assays, indicating the high quality of the screen results. A key outcome from this screen was the identification of *Atg8b* as a synthetic lethal partner of *NF1*.

Autophagy is a highly conserved mechanism for cellular degradation of unwanted or damaged cellular components. It is an important process for normal growth control; however, it can be defective under pathological conditions. Damaged organelles and unfolded proteins are sequestered into autophagosomes in the cytoplasm, which then undergo fusion with lysosomes (autolysosomes), resulting in degradation of the intracellular components [59]. The process of autophagy is tightly regulated by numerous proteins encoded by autophagy‐related genes (*ATGs*), including Atg8b (human ortholog: *GABARAP*), which is located in the autophagosome. Autophagic stimuli, including AMPK and mTOR signaling, can inhibit the mTORC1 complex, a negative regulator of autophagy. Following the dissociation of mTOR from ULK1, ULK1 Ser757 is dephosphorylated by PP2A [60] and PPM1D [61]. AMPK phosphorylates ULK1 at Ser 317 and Ser 777, enabling formation of the ULK1 complex and the subsequent induction of autophagy [62]. The phosphorylation of PIKfyve Ser1548 results in the conversion of PI to PI(5)P, which binds to WIPI2, resulting in separation of the isolation membrane from the endoplasmic reticulum, which will ultimately form the autophagosome [63]. Two ubiquitin systems regulate the formation of the double membrane bound autophagosome upon the binding of PI3KC3 via WIPI2: the ATG5/ATG16/ATG12 complex and the ATG8/LC3 system. Ubiquitination occurs via a positive feedback loop until the autophagosome is completely formed [64]. The autophagosome recruits lysosomal fusion proteins and ATGs are removed from the outer membrane. These vesicle fusion proteins, such as SNARE's and the Rab7 and PI3K III complex, enable fusion with the lysosome, forming autolysosomes [65, 66]. The cellular contents are subsequently degraded by the lysosomes and recycled.

In cancer, autophagy is reported to take on two opposing roles: (a) degradation of damaged organelles and recycling of macromolecules to maintain a stable cellular environment, which prevents the formation of tumors [67]; and (b) aiding in cancer cell survival in response to growth‐limiting conditions, contributing to tumorigenesis [68]. In the first reports of the tumor‐suppressive role of autophagy, allelic loss of BECN1 was identified in breast cancer cells and primary tumor material [69, 70]. Such impacts of autophagy dysregulation of tumor formation were subsequently found to be gene and tissue specific. Several studies have suggested that autophagy is regulated by tumor‐suppressive pathways, for example, p53 has been reported to modulate autophagy [71, 72]. Other studies support the theory of autophagy pathways as active tumor suppressors, such as those describing the allelic loss of BECN1 [69, 73]. More recently, two selective forms of autophagy have been linked to cancer: mitophagy and pexophagy. Mitophagy plays an important role in tumor suppression, as evidenced by the increase in reactive oxygen species and DNA damage due to the accumulation of damaged mitochondria in cells with the deletion of key autophagy genes [74]. In contrast to the role of mitophagy in cancer, the role of pexophagy, which mediates the removal of peroxisomes, is less well defined. Although it is widely accepted that autophagy suppresses tumor initiation, there is increasing evidence to suggest that autophagy is upregulated in established tumors. Support for this evidence was initially based on the finding that some tumors show increased levels of LC3 puncta and lapidated LC3 (LC3B), which indicates the accumulation of autophagosomes [75]. However, this static finding is only indicative of an increase in autophagic flux, a major limitation of studying autophagy in human cancer, as it is unable to distinguish between autophagosome turnover and the induction of autophagy. Regardless of this, multiple pre‐clinical studies have reported that autophagy supports the growth and metabolism of established tumors either via the activation of oncogenes, such as RAS, or the inactivation of tumor suppressors, such as p53 (reviewed in [76]). In addition, autophagy has been widely reported to promote cancer cell survival through a drug resistance mechanism [68, 77]. Therefore, targeting the autophagy pathway is a potential therapeutic option in the treatment of cancer.

Mutations in the *RAS* gene have been reported in 33% of human cancers [78]. RAS has previously been shown to modulate the basal levels of autophagy [79]. In addition, cancers associated with *RAS* mutations have been reported to be dependent on autophagy, although this appears to be tumor cell line dependent [80, 81]. Neurofibromin functions as a RASGAP (GTPase‐activating protein), which facilitates RAS inactivation by enabling its intrinsic GTPase activity [82]. In NF1‐associated tumors, neurofibromin expression is downregulated or absent, resulting in aberrant RAS activity and upregulation of the PI3K/AKT/mTORC1 pathway. Therefore, aberrant RAS activity is expected to negatively regulate autophagy; however, RAS is implicated in many signaling pathways and so it has a multifaceted role in autophagy regulation. RAS has been shown to negatively regulate starvation‐induced autophagy in *K‐RAS* G12V mutant NIH3T3 fibroblasts via the PI3K/AKT/mTORC1 pathway [83]. Furthermore, in a *Drosophila* development model where growth arrest and cell death where mediated in the salivary gland by autophagy, *RAS* G12V overexpression was shown to inhibit autophagy and salivary gland degeneration [84]. In contrast, mutant RAS has also been reported to activate autophagy. For example, H‐RAS G12V mutant NIH3T3 fibroblasts showed increased levels of autophagy via activation of the RAF1/MEK1/2/ERK pathway, subsequently inhibiting the binding of beclin 1 to BCL‐2 [85]. In addition, H‐RAS G12V was reported to promote autophagy in Rat2 fibroblasts through activation of the RAC1/MKK7/JNK signaling pathways, which upregulates ATG5 [86]. Furthermore, upregulation of ATG5 and ATG7 has been implicated in RAS‐induced autophagy and malignant cell transformation [86, 87]. Therefore, RAS can promote or suppress autophagy by affecting numerous signaling pathways.

Autophagy is a complex process involving many different genes. In addition to *Atg8b*, *Uba1*, an additional autophagy‐related gene, was identified in the screen [88]. While the selective effects of inhibiting autophagy were consistent and reproducible in secondary assays, it was surprising that more autophagy linked genes were not identified in our screens. Upon further investigation, we found that approximately half of the 30 autophagy genes tested with low throughput assays showed the selective effects observed with *Atg8b*. These genes have roles from early‐stage vesicle induction to late‐stage fusion of the autophagosome and lysosome. These results suggest that the selective effects are not caused by disruption of an isolated step in the autophagy pathway but by general perturbation across multiple steps. It is likely that other autophagy genes were missed by the screen due to ineffective RNAi reagents or noise.

Interestingly, *Atg5*, which is involved in the induction of autophagy via autophagic vesicle formation, was found to have the most significant synthetic lethal interaction with *NF1*. Previous studies have implicated ATG5 upregulation in RAS‐induced autophagy and malignant cell transformation [86, 87]. However, at present, there are no commercially available inhibitors of ATG5. Further studies are required to determine the exact autophagy genes that are dysregulated in our panel of human *NF1*‐deficient cell lines.

In total, we assessed seven drugs known to inhibit autophagy, and all reproduced the selective effects in *NF1*‐deficient cells. However, there was some variation in effects with some drugs not being effective across all cell lines tested. CQ appears to be the most promising of the drugs that we tested for clinical application. It is already approved for clinical use and produced the most consistent and robust effects across the different NF1 cell models that we tested. CQ is a widely used anti‐malarial that functions to inhibit the binding of autophagosomes to lysosomes by diffusing into the lysosomes and altering the acidic environment, thereby inhibiting autophagic lysosomal degradation [36]. Previous studies have shown that CQ and its derivative hydroxychloroquine have anti‐tumor properties in several types of cancer, including glioblastoma [89, 90], hepatocellular carcinoma [91], prostate cancer [92], breast cancer [93], and pancreatic cancer [94]. In addition, a recent study reported that the expression of metalloproteinase 1 (MMP1) was down‐regulated in *NF1*‐deficient fibroblasts, with a further reduction associated with lysosomal degradation of MMP1. Interestingly, treatment of *NF1*‐deficient cells with CQ restored MMP1 expression via two mechanisms: activation of the AHR/ERK pathway to enhance the mRNA and protein expression of MMP1, and inhibition of the lysosomal degradation of MMP1 [95]. Although this study did not assess whether autophagy was dysregulated in the *NF1*‐deficient fibroblasts, Schwann cells, or cells derived from NF1 tumors, it does highlight the therapeutic potential of autophagy inhibition. Similarly, we found that CQ significantly reduced dNF1‐KO cell viability relative to wild‐type S2R+ cells across a range of concentrations under conditions where autophagy is induced (serum starvation of cells and *NF1* deficiency). This effect of CQ was conserved across a panel of three human *NF1*‐deficient cell lines, two of which were derived from plexiform neurofibromas from NF1 patients. In addition, the effects of CQ on cell viability were found to be more potent and selective than selumetinib. Furthermore, CQ altered the survival of *dNf1*‐deficient *Drosophila in vivo* and reduced the growth of *NF1*‐mutant xenografts in mice to an even greater extent than selumetinib. The doses of CQ and selumetinib administered to the mice were deemed non‐toxic, as has been widely reported previously [96, 97]. Through the inhibition of autophagy with CQ, we observed an increased level of apoptosis in the CQ‐treated xenografts, as assessed with the levels of cleaved PARP. Therefore, CQ shows great potential as a therapeutic agent for NF1‐associated tumors.

While there is a vast array of evidence for the efficacy and safety of CQ, the underlying mechanisms of the tumor suppressive actions of CQ remain to be determined. One potential mechanism is that under starvation conditions, such as those used in this study, a reduction in glucose transport results in a release of mTOR inhibition of the ULK1 complex, inducing vesicle nucleation and facilitating the process of autophagy. Inhibition of the lysosome using CQ has been shown to inhibit tumor growth and induce tumor cell death *in vitro* [98, 99]. We speculate that *NF1*‐deficient cells are more susceptible to autophagy inhibition with CQ because the baseline levels of autophagic flux are higher, as shown in Figs 3 and 5. Aberrant RAS activity has previously been shown to regulate autophagic flux [79], which may result in such cancers being more susceptible to autophagy inhibition with CQ. Therefore, we speculate that aberrant RAS activity in *NF1*‐deficient cells results in the initiation of autophagy, and that inhibition of this pathway has anti‐tumor effects. However, we also acknowledge that we observed no difference in the phosphorylation of ERK and AKT between S2R+/dNF1‐KO cells and WT/C8/C23 Schwann cells under baseline and serum free conditions, which suggests that RAS activity levels are similar in the two cell lines. Therefore, while there is extensive evidence that RAS activation leads to dependence on autophagy, it is possible that there is a second, RAS‐independent, mechanism that also contributes to the selective effect of autophagy inhibition. In addition, we acknowledge that there is a technical possibility that the effects of CQ are independent of the selective effects on autophagy. Regardless of the mechanism, when combining our data on the effects of CQ and bafilomycin A1, we provide strong evidence for inhibition of the autophagy pathway as a target in the treatment of NF1‐associated tumors.

Finally, we found that inhibition of autophagy with CQ and bafilomycin A1 increased the sensitivity of some *NF1*‐deficient cells to MEK1/2 inhibition with selumetinib. This finding is in line with a previous study, which showed that combined inhibition of MEK1/2 plus autophagy had a synergistic anti‐proliferative effect in pancreatic ductal adenocarcinoma cell lines, which display aberrant K‐RAS activity, as well as patient‐derived pancreatic ductal adenocarcinoma xenograft tumors in mice [100]. Selumetinib is currently the only FDA‐approved drug for the treatment of tumors associated with neurofibromatosis type 1 [55]. The phase 2 trial (SPRINT) for the use of selumetinib in PNs reported clinically meaningful improvements in 71% of patients [10], prompting its FDA approval for patients ages 2 to 18 years with NF1 who have symptomatic, inoperable PNs. However, there are toxicity effects related to long‐term MEK inhibition.

## 5 Conclusions

In conclusion, we have shown using multiple techniques, reagents, and models that inhibition of autophagy has potential as a novel therapeutic strategy for the treatment of NF1 tumors. Given the existing clinical use of CQ and the robust and conserved effects that we observe between cell culture models, this candidate drug has a high chance of successful translation to the clinic, resulting in a positive impact on NF1 patients.

## Conflict of interest

BEH was a shareholder and founding director of Quest Genetics Ltd between 2021 and 2024. The remaining authors declare no competing interests.

## Author contributions

MS, NP, JAW and BEH conceived and designed the experiments, MS, YW, SJB, TRM, AS, SS and AH performed the experiments and acquired the data, MS, YW, SJB, TRM, AS, SS, AH, JAW and BEH analyzed and interpreted the data, MS, SJB, JAW, and BEH wrote the paper.

### Peer review

The peer review history for this article is available at https://www.webofscience.com/api/gateway/wos/peer‐review/10.1002/1878‐0261.13704.

## Supporting information


**Fig. S1.** Identification of synthetic lethal interactions with *NF1*.
**Fig. S2.** The NF1 gene is well conserved between *Drosophila* and humans with 68% identity at the amino acid level.
**Fig. S3.** Western blot data.
**Fig. S4.** Drug dose curves in *Drosophila* cells.
**Fig. S5.** Autophagy pathway activity in NF1‐deficient and control cells.
**Fig. S6.** Drug dose curves of autophagy inhibitors in human cells.
**Fig. S7.** CQ results in an increased dose‐dependent lethality of *dNf1* ^*C1*^ mutants compared to WT control flies.
**Fig. S8.** Mouse weight change during treatment.

**Table S1.** Results from the genome wide synthetic lethal interaction screen in *Drosophila* cells.

**Table S2.** *Drosophila* genes identified as having synthetic lethal interactions with *NF1* and their human orthologs.

**Table S3.** The 29 autophagy genes screened for synthetic lethal interactions with *NF1* using VDA analysis.

## Data Availability

The data supporting the findings of this study are available in the figure, tables and supplementary material of this article.

## References

[1] Evans DG , Howard E , Giblin C , Clancy T , Spencer H , Huson SM , et al. Birth incidence and prevalence of tumor‐prone syndromes: estimates from a UK family genetic register service. Am J Med Genet A. 2010;152A(2):327–332. 20082463 10.1002/ajmg.a.33139

[2] Gutmann DH , Ferner RE , Listernick RH , Korf BR , Wolters PL , Johnson KJ . Neurofibromatosis type 1. Nat Rev Dis Primers. 2017;3:17004. 28230061 10.1038/nrdp.2017.4

[3] Lin AL , Gutmann DH . Advances in the treatment of neurofibromatosis‐associated tumours. Nat Rev Clin Oncol. 2013;10(11):616–624. 23939548 10.1038/nrclinonc.2013.144

[4] Rasmussen SA , Yang Q , Friedman JM . Mortality in neurofibromatosis 1: an analysis using U.S. death certificates. Am J Hum Genet. 2001;68(5):1110–1118. 11283797 10.1086/320121 PMC1226092

[5] Martin GA , Viskochil D , Bollag G , McCabe PC , Crosier WJ , Haubruck H , et al. The GAP‐related domain of the neurofibromatosis type 1 gene product interacts with ras p21. Cell. 1990;63(4):843–849. 2121370 10.1016/0092-8674(90)90150-d

[6] Xu GF , Lin B , Tanaka K , Dunn D , Wood D , Gesteland R , et al. The catalytic domain of the neurofibromatosis type 1 gene product stimulates ras GTPase and complements ira mutants of S. Cerevisiae. Cell. 1990;63(4):835–841. 2121369 10.1016/0092-8674(90)90149-9

[7] Viskochil D , White R , Cawthon R . The neurofibromatosis type 1 gene. Annu Rev Neurosci. 1993;16:183–205. 8460890 10.1146/annurev.ne.16.030193.001151

[8] Ratner N , Miller SJ . A RASopathy gene commonly mutated in cancer: the neurofibromatosis type 1 tumour suppressor. Nat Rev Cancer. 2015;15(5):290–301. 25877329 10.1038/nrc3911 PMC4822336

[9] Simanshu DK , Nissley DV , McCormick F . RAS proteins and their regulators in human disease. Cell. 2017;170(1):17–33. 28666118 10.1016/j.cell.2017.06.009 PMC5555610

[10] Gross AM , Wolters PL , Dombi E , Baldwin A , Whitcomb P , Fisher MJ , et al. Selumetinib in children with inoperable plexiform Neurofibromas. N Engl J Med. 2020;382(15):1430–1442. 32187457 10.1056/NEJMoa1912735 PMC7305659

[11] Abdel‐Rahman O , ElHalawani H , Ahmed H . Risk of selected cardiovascular toxicities in patients with cancer treated with MEK inhibitors: a comparative systematic review and meta‐analysis. J Glob Oncol. 2015;1(2):73–82. 28804776 10.1200/JGO.2015.000802 PMC5539872

[12] Avery RA , Trimboli‐Heidler C , Kilburn LB . Separation of outer retinal layers secondary to selumetinib. J AAPOS. 2016;20(3):268–271. 27108842 10.1016/j.jaapos.2016.01.012 PMC4912405

[13] Baldo F , Magnolato A , Barbi E , Bruno I . Selumetinib side effects in children treated for plexiform neurofibromas: first case reports of peripheral edema and hair color change. BMC Pediatr. 2021;21(1):67. 33549085 10.1186/s12887-021-02530-5 PMC7866429

[14] Anderson MK , Johnson M , Thornburg L , Halford Z . A review of selumetinib in the treatment of neurofibromatosis type 1‐related plexiform neurofibromas. Ann Pharmacother. 2022;56(6):716–726. 34541874 10.1177/10600280211046298

[15] Kaelin WG Jr . The concept of synthetic lethality in the context of anticancer therapy. Nat Rev Cancer. 2005;5(9):689–698. 16110319 10.1038/nrc1691

[16] Lord CJ , Ashworth A . PARP inhibitors: synthetic lethality in the clinic. Science. 2017;355(6330):1152–1158. 28302823 10.1126/science.aam7344 PMC6175050

[17] Ryan CJ , Bajrami I , Lord CJ . Synthetic lethality and cancer – penetrance as the major barrier. Trends Cancer. 2018;4(10):671–683. 30292351 10.1016/j.trecan.2018.08.003

[18] Valvezan AJ , Turner M , Belaid A , Lam HC , Miller SK , McNamara MC , et al. mTORC1 couples nucleotide synthesis to nucleotide demand resulting in a targetable metabolic vulnerability. Cancer Cell. 2017;32(5):624–638.e5. 29056426 10.1016/j.ccell.2017.09.013 PMC5687294

[19] Nicholson HE , Tariq Z , Housden BE , Jennings RB , Stransky LA , Perrimon N , et al. HIF‐independent synthetic lethality between CDK4/6 inhibition and VHL loss across species. Sci Signal. 2019;12(601):eaay0482. 31575731 10.1126/scisignal.aay0482 PMC6913182

[20] Housden BE , Valvezan AJ , Kelley C , Sopko R , Hu Y , Roesel C , et al. Identification of potential drug targets for tuberous sclerosis complex by synthetic screens combining CRISPR‐based knockouts with RNAi. Sci Signal. 2015;8(393):rs9. 26350902 10.1126/scisignal.aab3729 PMC4642709

[21] Li H , Chang LJ , Neubauer DR , Muir DF , Wallace MR . Immortalization of human normal and NF1 neurofibroma Schwann cells. Lab Investig. 2016;96(10):1105–1115. 27617404 10.1038/labinvest.2016.88

[22] Housden BE , Nicholson HE , Perrimon N . Synthetic lethality screens using RNAi in combination with CRISPR‐based knockout in *Drosophila* cells. Bio Protoc. 2017;7(3):e2119. 10.21769/BioProtoc.2119 PMC5432019 28523286

[23] Shalem O , Sanjana NE , Hartenian E , Shi X , Scott DA , Mikkelson T , et al. Genome‐scale CRISPR‐Cas9 knockout screening in human cells. Science. 2014;343(6166):84–87. 24336571 10.1126/science.1247005 PMC4089965

[24] Housden BE , Li Z , Kelley C , Wang Y , Hu Y , Valvezan AJ , et al. Improved detection of synthetic lethal interactions in *Drosophila* cells using variable dose analysis (VDA). Proc Natl Acad Sci USA. 2017;114(50):E10755–E10762. 29183982 10.1073/pnas.1713362114 PMC5740648

[25] Sierzputowska K , Baxter CR , Housden BE . Variable dose analysis: a novel high‐throughput RNAi screening method for *Drosophila* cells. Bio Protoc. 2018;8(24):e3112. 10.21769/BioProtoc.3112 PMC8342101 34532554

[26] Botero V , Stanhope BA , Brown EB , Grenci EC , Boto T , Park SJ , et al. Neurofibromin regulates metabolic rate via neuronal mechanisms in *Drosophila* . Nat Commun. 2021;12(1):4285. 34257279 10.1038/s41467-021-24505-x PMC8277851

[27] The I , Hannigan GE , Cowley GS , Reginald S , Zhong Y , Gusella JF , et al. Rescue of a *Drosophila* NF1 mutant phenotype by protein kinase a. Science. 1997;276(5313):791–794. 9115203 10.1126/science.276.5313.791

[28] Ren X , Sun J , Housden BE , Hu Y , Roesel C , Lin S , et al. Optimized gene editing technology for *Drosophila melanogaster* using germ line‐specific Cas9. Proc Natl Acad Sci USA. 2013;110(47):19012–19017. 24191015 10.1073/pnas.1318481110 PMC3839733

[29] Walker JA , Tchoudakova AV , McKenney PT , Brill S , Wu D , Cowley GS , et al. Reduced growth of *Drosophila* neurofibromatosis 1 mutants reflects a non‐cell‐autonomous requirement for GTPase‐activating protein activity in larval neurons. Genes Dev. 2006;20(23):3311–3323. 17114577 10.1101/gad.1466806 PMC1686607

[30] Dietzl G , Chen D , Schnorrer F , Su KC , Barinova Y , Fellner M , et al. A genome‐wide transgenic RNAi library for conditional gene inactivation in *Drosophila* . Nature. 2007;448(7150):151–156. 17625558 10.1038/nature05954

[31] Lee H , McManus CJ , Cho DY , Eaton M , Renda F , Somma MP , et al. DNA copy number evolution in *Drosophila* cell lines. Genome Biol. 2014;15(8):R70. 25262759 10.1186/gb-2014-15-8-r70 PMC4289277

[32] Ashton‐Beaucage D , Udell CM , Gendron P , Sahmi M , Lefrancois M , Baril C , et al. A functional screen reveals an extensive layer of transcriptional and splicing control underlying RAS/MAPK signaling in *Drosophila* . PLoS Biol. 2014;12(3):e1001809. 24643257 10.1371/journal.pbio.1001809 PMC3958334

[33] Friedman A , Perrimon N . A functional RNAi screen for regulators of receptor tyrosine kinase and ERK signalling. Nature. 2006;444(7116):230–234. 17086199 10.1038/nature05280

[34] Szklarczyk D , Gable AL , Lyon D , Junge A , Wyder S , Huerta‐Cepas J , et al. STRING v11: protein‐protein association networks with increased coverage, supporting functional discovery in genome‐wide experimental datasets. Nucleic Acids Res. 2019;47(D1):D607–D613. 30476243 10.1093/nar/gky1131 PMC6323986

[35] Zhou W , Wang H , Yang Y , Chen ZS , Zou C , Zhang J . Chloroquine against malaria, cancers and viral diseases. Drug Discov Today. 2020;25(11):2012–2022. 32947043 10.1016/j.drudis.2020.09.010 PMC7492153

[36] Homewood CA , Warhurst DC , Peters W , Baggaley VC . Lysosomes, pH and the anti‐malarial action of chloroquine. Nature. 1972;235(5332):50–52. 4550396 10.1038/235050a0

[37] Ursing J , Rombo L , Eksborg S , Larson L , Bruvoll A , Tarning J , et al. High‐dose chloroquine for uncomplicated *Plasmodium falciparum* malaria is well tolerated and causes similar QT interval prolongation as standard‐dose chloroquine in children. Antimicrob Agents Chemother. 2020;64(3):e01846‐19. 31907183 10.1128/AAC.01846-19 PMC7038251

[38] Levy JMM , Towers CG , Thorburn A . Targeting autophagy in cancer. Nat Rev Cancer. 2017;17(9):528–542. 28751651 10.1038/nrc.2017.53 PMC5975367

[39] Petherick KJ , Conway OJ , Mpamhanga C , Osborne SA , Kamal A , Saxty B , et al. Pharmacological inhibition of ULK1 kinase blocks mammalian target of rapamycin (mTOR)‐dependent autophagy. J Biol Chem. 2015;290(18):11376–11383. 25833948 10.1074/jbc.C114.627778 PMC4416842

[40] Bago R , Malik N , Munson MJ , Prescott AR , Davies P , Sommer E , et al. Characterization of VPS34‐IN1, a selective inhibitor of Vps34, reveals that the phosphatidylinositol 3‐phosphate‐binding SGK3 protein kinase is a downstream target of class III phosphoinositide 3‐kinase. Biochem J. 2014;463(3):413–427. 25177796 10.1042/BJ20140889 PMC4209782

[41] Akin D , Wang SK , Habibzadegah‐Tari P , Law B , Ostrov D , Li M , et al. A novel ATG4B antagonist inhibits autophagy and has a negative impact on osteosarcoma tumors. Autophagy. 2014;10(11):2021–2035. 25483883 10.4161/auto.32229 PMC4502682

[42] Bosc D , Vezenkov L , Bortnik S , An J , Xu J , Choutka C , et al. A new quinoline‐based chemical probe inhibits the autophagy‐related cysteine protease ATG4B. Sci Rep. 2018;8(1):11653. 30076329 10.1038/s41598-018-29900-x PMC6076261

[43] Zhang L , Qiang P , Yu J , Miao Y , Chen Z , Qu J , et al. Identification of compound CA‐5f as a novel late‐stage autophagy inhibitor with potent anti‐tumor effect against non‐small cell lung cancer. Autophagy. 2019;15(3):391–406. 30145925 10.1080/15548627.2018.1511503 PMC6351124

[44] Koehler BC , Jassowicz A , Scherr AL , Lorenz S , Radhakrishnan P , Kautz N , et al. Pan‐Bcl‐2 inhibitor obatoclax is a potent late stage autophagy inhibitor in colorectal cancer cells independent of canonical autophagy signaling. BMC Cancer. 2015;15:919. 26585594 10.1186/s12885-015-1929-y PMC4653869

[45] Mauvezin C , Neufeld TP . Bafilomycin A1 disrupts autophagic flux by inhibiting both V‐ATPase‐dependent acidification and Ca‐P60A/SERCA‐dependent autophagosome‐lysosome fusion. Autophagy. 2015;11(8):1437–1438. 26156798 10.1080/15548627.2015.1066957 PMC4590655

[46] Kocaturk NM , Akkoc Y , Kig C , Bayraktar O , Gozuacik D , Kutlu O . Autophagy as a molecular target for cancer treatment. Eur J Pharm Sci. 2019;134:116–137. 30981885 10.1016/j.ejps.2019.04.011

[47] Wang W , Cui XW , Gu YH , Wei CJ , Li YH , Ren JY , et al. Combined cyclin‐dependent kinase inhibition overcomes MAPK/extracellular signal‐regulated kinase kinase inhibitor resistance in plexiform neurofibroma of neurofibromatosis type I. J Invest Dermatol. 2022;142(3 Pt A):613–623.e7. 34534577 10.1016/j.jid.2021.07.164

[48] Tian Z , You Y , Xiao M , Liu J , Xu G , Ma C , et al. Inhibition of YAP sensitizes the selumetinib treatment for neurofibromatosis type 1 related plexiform neurofibroma. Int J Med Sci. 2023;20(1):125–135. 36619222 10.7150/ijms.78386 PMC9812799

[49] King LB , Boto T , Botero V , Aviles AM , Jomsky BM , Joseph C , et al. Developmental loss of neurofibromin across distributed neuronal circuits drives excessive grooming in *Drosophila* . PLoS Genet. 2020;16(7):e1008920. 32697780 10.1371/journal.pgen.1008920 PMC7398555

[50] Moscato EH , Dubowy C , Walker JA , Kayser MS . Social behavioral deficits with loss of neurofibromin emerge from peripheral chemosensory neuron dysfunction. Cell Rep. 2020;32(1):107856. 32640222 10.1016/j.celrep.2020.107856 PMC7416787

[51] Bai L , Lee Y , Hsu CT , Williams JA , Cavanaugh D , Zheng X , et al. A conserved circadian function for the neurofibromatosis 1 gene. Cell Rep. 2018;22(13):3416–3426. 29590612 10.1016/j.celrep.2018.03.014 PMC5898822

[52] Walker JA , Gouzi JY , Long JB , Huang S , Maher RC , Xia H , et al. Genetic and functional studies implicate synaptic overgrowth and ring gland cAMP/PKA signaling defects in the *Drosophila melanogaster* neurofibromatosis‐1 growth deficiency. PLoS Genet. 2013;9(11):e1003958. 24278035 10.1371/journal.pgen.1003958 PMC3836801

[53] Gouzi JY , Moressis A , Walker JA , Apostolopoulou AA , Palmer RH , Bernards A , et al. The receptor tyrosine kinase Alk controls neurofibromin functions in *Drosophila* growth and learning. PLoS Genet. 2011;7(9):e1002281. 21949657 10.1371/journal.pgen.1002281 PMC3174217

[54] Liao CP , Pradhan S , Chen Z , Patel AJ , Booker RC , Le LQ . The role of nerve microenvironment for neurofibroma development. Oncotarget. 2016;7(38):61500–61508. 27517146 10.18632/oncotarget.11133 PMC5308667

[55] Markham A , Keam SJ . Selumetinib: first approval. Drugs. 2020;80(9):931–937. 32504375 10.1007/s40265-020-01331-x

[56] Chen L , Ye HL , Zhang G , Yao WM , Chen XZ , Zhang FC , et al. Autophagy inhibition contributes to the synergistic interaction between EGCG and doxorubicin to kill the hepatoma Hep3B cells. PLoS One. 2014;9(1):e85771. 24465696 10.1371/journal.pone.0085771 PMC3897495

[57] Liu B , Huang X , Hu Y , Chen T , Peng B , Gao N , et al. Ethacrynic acid improves the antitumor effects of irreversible epidermal growth factor receptor tyrosine kinase inhibitors in breast cancer. Oncotarget. 2016;7(36):58038–58050. 27487128 10.18632/oncotarget.10846 PMC5295410

[58] Sagwal SK , Pasqual‐Melo G , Bodnar Y , Gandhirajan RK , Bekeschus S . Combination of chemotherapy and physical plasma elicits melanoma cell death via upregulation of SLC22A16. Cell Death Dis. 2018;9(12):1179. 30518936 10.1038/s41419-018-1221-6 PMC6281583

[59] Zhang XJ , Chen S , Huang KX , Le WD . Why should autophagic flux be assessed? Acta Pharmacol Sin. 2013;34(5):595–599. 23474710 10.1038/aps.2012.184 PMC4002868

[60] Wong PM , Feng Y , Wang J , Shi R , Jiang X . Regulation of autophagy by coordinated action of mTORC1 and protein phosphatase 2A. Nat Commun. 2015;6:8048. 26310906 10.1038/ncomms9048 PMC4552084

[61] Torii S , Yoshida T , Arakawa S , Honda S , Nakanishi A , Shimizu S . Identification of PPM1D as an essential Ulk1 phosphatase for genotoxic stress‐induced autophagy. EMBO Rep. 2016;17(11):1552–1564. 27670885 10.15252/embr.201642565 PMC5090708

[62] Kim J , Kundu M , Viollet B , Guan KL . AMPK and mTOR regulate autophagy through direct phosphorylation of Ulk1. Nat Cell Biol. 2011;13(2):132–141. 21258367 10.1038/ncb2152 PMC3987946

[63] Karabiyik C , Vicinanza M , Son SM , Rubinsztein DC . Glucose starvation induces autophagy via ULK1‐mediated activation of PIKfyve in an AMPK‐dependent manner. Dev Cell. 2021;56(13):1961–1975.e5. 34107300 10.1016/j.devcel.2021.05.010

[64] Fracchiolla D , Chang C , Hurley JH , Martens S . A PI3K‐WIPI2 positive feedback loop allosterically activates LC3 lipidation in autophagy. J Cell Biol. 2020;219(7):e201912098. 32437499 10.1083/jcb.201912098 PMC7337497

[65] Moreau K , Renna M , Rubinsztein DC . Connections between SNAREs and autophagy. Trends Biochem Sci. 2013;38(2):57–63. 23306003 10.1016/j.tibs.2012.11.004

[66] Zhong Y , Wang QJ , Li X , Yan Y , Backer JM , Chait BT , et al. Distinct regulation of autophagic activity by Atg14L and rubicon associated with Beclin 1‐phosphatidylinositol‐3‐kinase complex. Nat Cell Biol. 2009;11(4):468–476. 19270693 10.1038/ncb1854 PMC2664389

[67] Mizushima N , Levine B , Cuervo AM , Klionsky DJ . Autophagy fights disease through cellular self‐digestion. Nature. 2008;451(7182):1069–1075. 18305538 10.1038/nature06639 PMC2670399

[68] O'Donovan TR , O'Sullivan GC , McKenna SL . Induction of autophagy by drug‐resistant esophageal cancer cells promotes their survival and recovery following treatment with chemotherapeutics. Autophagy. 2011;7(5):509–524. 21325880 10.4161/auto.7.6.15066 PMC3127212

[69] Liang XH , Jackson S , Seaman M , Brown K , Kempkes B , Hibshoosh H , et al. Induction of autophagy and inhibition of tumorigenesis by beclin 1. Nature. 1999;402(6762):672–676. 10604474 10.1038/45257

[70] Enzalutamide. Bethesda (MD) National Institute of Diabetes and Digestive and Kidney Diseases LiverTox: Clinical and Research Information on Drug‐Induced Liver Injury. https://wwwncbinlmnihgov/books/NBK548070/2012. 31643176

[71] Tasdemir E , Maiuri MC , Galluzzi L , Vitale I , Djavaheri‐Mergny M , D'Amelio M , et al. Regulation of autophagy by cytoplasmic p53. Nat Cell Biol. 2008;10(6):676–687. 18454141 10.1038/ncb1730 PMC2676564

[72] Crighton D , Wilkinson S , O'Prey J , Syed N , Smith P , Harrison PR , et al. DRAM, a p53‐induced modulator of autophagy, is critical for apoptosis. Cell. 2006;126(1):121–134. 16839881 10.1016/j.cell.2006.05.034

[73] Aita VM , Liang XH , Murty VV , Pincus DL , Yu W , Cayanis E , et al. Cloning and genomic organization of beclin 1, a candidate tumor suppressor gene on chromosome 17q21. Genomics. 1999;59(1):59–65. 10395800 10.1006/geno.1999.5851

[74] Poole LP , Macleod KF . Mitophagy in tumorigenesis and metastasis. Cell Mol Life Sci. 2021;78(8):3817–3851. 33580835 10.1007/s00018-021-03774-1 PMC8259496

[75] Fujii S , Mitsunaga S , Yamazaki M , Hasebe T , Ishii G , Kojima M , et al. Autophagy is activated in pancreatic cancer cells and correlates with poor patient outcome. Cancer Sci. 2008;99(9):1813–1819. 18616529 10.1111/j.1349-7006.2008.00893.x PMC11159933

[76] Debnath J , Gammoh N , Ryan KM . Autophagy and autophagy‐related pathways in cancer. Nat Rev Mol Cell Biol. 2023;24(8):560–575. 36864290 10.1038/s41580-023-00585-z PMC9980873

[77] Peng X , Gong F , Chen Y , Jiang Y , Liu J , Yu M , et al. Autophagy promotes paclitaxel resistance of cervical cancer cells: involvement of Warburg effect activated hypoxia‐induced factor 1‐alpha‐mediated signaling. Cell Death Dis. 2014;5:e1367. 25118927 10.1038/cddis.2014.297 PMC4454295

[78] Karnoub AE , Weinberg RA . Ras oncogenes: split personalities. Nat Rev Mol Cell Biol. 2008;9(7):517–531. 18568040 10.1038/nrm2438 PMC3915522

[79] Schmukler E , Kloog Y , Pinkas‐Kramarski R . Ras and autophagy in cancer development and therapy. Oncotarget. 2014;5(3):577–586. 24583697 10.18632/oncotarget.1775 PMC3996671

[80] Guo JY , Chen HY , Mathew R , Fan J , Strohecker AM , Karsli‐Uzunbas G , et al. Activated Ras requires autophagy to maintain oxidative metabolism and tumorigenesis. Genes Dev. 2011;25(5):460–470. 21317241 10.1101/gad.2016311 PMC3049287

[81] Morgan MJ , Gamez G , Menke C , Hernandez A , Thorburn J , Gidan F , et al. Regulation of autophagy and chloroquine sensitivity by oncogenic RAS in vitro is context‐dependent. Autophagy. 2014;10(10):1814–1826. 25136801 10.4161/auto.32135 PMC4198365

[82] Downward J . Targeting RAS signalling pathways in cancer therapy. Nat Rev Cancer. 2003;3(1):11–22. 12509763 10.1038/nrc969

[83] Furuta S , Hidaka E , Ogata A , Yokota S , Kamata T . Ras is involved in the negative control of autophagy through the class I PI3‐kinase. Oncogene. 2004;23(22):3898–3904. 15064741 10.1038/sj.onc.1207539

[84] Berry DL , Baehrecke EH . Growth arrest and autophagy are required for salivary gland cell degradation in *Drosophila* . Cell. 2007;131(6):1137–1148. 18083103 10.1016/j.cell.2007.10.048 PMC2180345

[85] Wu SY , Lan SH , Cheng DE , Chen WK , Shen CH , Lee YR , et al. Ras‐related tumorigenesis is suppressed by BNIP3‐mediated autophagy through inhibition of cell proliferation. Neoplasia. 2011;13(12):1171–1182. 22241963 10.1593/neo.11888 PMC3257192

[86] Byun JY , Yoon CH , An S , Park IC , Kang CM , Kim MJ , et al. The Rac1/MKK7/JNK pathway signals upregulation of Atg5 and subsequent autophagic cell death in response to oncogenic Ras. Carcinogenesis. 2009;30(11):1880–1888. 19783847 10.1093/carcin/bgp235

[87] Kim MJ , Woo SJ , Yoon CH , Lee JS , An S , Choi YH , et al. Involvement of autophagy in oncogenic K‐Ras‐induced malignant cell transformation. J Biol Chem. 2011;286(15):12924–12932. 21300795 10.1074/jbc.M110.138958 PMC3075639

[88] Chang TK , Shravage BV , Hayes SD , Powers CM , Simin RT , Wade Harper J , et al. Uba1 functions in Atg7‐ and Atg3‐independent autophagy. Nat Cell Biol. 2013;15(9):1067–1078. 23873149 10.1038/ncb2804 PMC3762904

[89] Golden EB , Cho HY , Jahanian A , Hofman FM , Louie SG , Schonthal AH , et al. Chloroquine enhances temozolomide cytotoxicity in malignant gliomas by blocking autophagy. Neurosurg Focus. 2014;37(6):E12. 10.3171/2014.9.FOCUS14504 25434381

[90] Zanotto‐Filho A , Braganhol E , Klafke K , Figueiro F , Terra SR , Paludo FJ , et al. Autophagy inhibition improves the efficacy of curcumin/temozolomide combination therapy in glioblastomas. Cancer Lett. 2015;358(2):220–231. 25542083 10.1016/j.canlet.2014.12.044

[91] Mei L , Chen Y , Wang Z , Wang J , Wan J , Yu C , et al. Synergistic anti‐tumour effects of tetrandrine and chloroquine combination therapy in human cancer: a potential antagonistic role for p21. Br J Pharmacol. 2015;172(9):2232–2245. 25521075 10.1111/bph.13045 PMC4403090

[92] Farrow JM , Yang JC , Evans CP . Autophagy as a modulator and target in prostate cancer. Nat Rev Urol. 2014;11(9):508–516. 25134829 10.1038/nrurol.2014.196 PMC4415606

[93] Lefort S , Joffre C , Kieffer Y , Givel AM , Bourachot B , Zago G , et al. Inhibition of autophagy as a new means of improving chemotherapy efficiency in high‐LC3B triple‐negative breast cancers. Autophagy. 2014;10(12):2122–2142. 25427136 10.4161/15548627.2014.981788 PMC4502743

[94] Yang A , Kimmelman AC . Inhibition of autophagy attenuates pancreatic cancer growth independent of TP53/TRP53 status. Autophagy. 2014;10(9):1683–1684. 25046107 10.4161/auto.29961 PMC4206544

[95] Tsuji G , Takai‐Yumine A , Kato T , Furue M . Metalloproteinase 1 downregulation in neurofibromatosis 1: therapeutic potential of antimalarial hydroxychloroquine and chloroquine. Cell Death Dis. 2021;12(6):513. 34011935 10.1038/s41419-021-03802-9 PMC8134427

[96] Tang MC , Wu MY , Hwang MH , Chang YT , Huang HJ , Lin AM , et al. Chloroquine enhances gefitinib cytotoxicity in gefitinib‐resistant nonsmall cell lung cancer cells. PLoS One. 2015;10(3):e0119135. 25807554 10.1371/journal.pone.0119135 PMC4373825

[97] Troiani T , Vecchione L , Martinelli E , Capasso A , Costantino S , Ciuffreda LP , et al. Intrinsic resistance to selumetinib, a selective inhibitor of MEK1/2, by cAMP‐dependent protein kinase a activation in human lung and colorectal cancer cells. Br J Cancer. 2012;106(10):1648–1659. 22569000 10.1038/bjc.2012.129 PMC3349172

[98] Eng CH , Wang Z , Tkach D , Toral‐Barza L , Ugwonali S , Liu S , et al. Macroautophagy is dispensable for growth of KRAS mutant tumors and chloroquine efficacy. Proc Natl Acad Sci USA. 2016;113(1):182–187. 26677873 10.1073/pnas.1515617113 PMC4711870

[99] Jia L , Wang J , Wu T , Wu J , Ling J , Cheng B . In vitro and in vivo antitumor effects of chloroquine on oral squamous cell carcinoma. Mol Med Rep. 2017;16(5):5779–5786. 28849182 10.3892/mmr.2017.7342 PMC5865757

[100] Kinsey CG , Camolotto SA , Boespflug AM , Guillen KP , Foth M , Truong A , et al. Protective autophagy elicited by RAF→MEK→ERK inhibition suggests a treatment strategy for RAS‐driven cancers. Nat Med. 2019;25(4):620–627. 30833748 10.1038/s41591-019-0367-9 PMC6452642

[101] Shannon P , Markiel A , Ozier O , Baliga NS , Wang JT , Ramage D , et al. Cytoscape: a software environment for integrated models of biomolecular interaction networks. Genome Res. 2003;13(11):2498–2504. 14597658 10.1101/gr.1239303 PMC403769

