# Correction to ‘Inhibition of autophagy as a novel treatment for neurofibromatosis type 1 tumors’

**DOI:** 10.1002/1878-0261.70162

**Published:** 2025-11-14

Stevens M, Wang Y, Bouley SJ, Mandigo TR, Sharma A, Sengupta S, Housden A, Perrimon N, Walker JA, Housden BE. Inhibition of autophagy as a novel treatment for neurofibromatosis type 1 tumors. *Mol Oncol*. 2025;19(3):825–851. https://doi.org/10.1002/1878-0261.13704.

In the manuscript by Stevens et al. (2025), the authors would like to correct the authorship list by including ‘Sebastian Oltean’ as an additional author in the 8th position. The amended list of authors would therefore be: Stevens M, Wang Y, Bouley SJ, Mandigo TR, Sharma A, Sengupta S, Housden A, **Oltean S**, Perrimon N, Walker JA, Housden BE.

The authors would also like to update the ‘Author Contributions’ section to read:

MS, NP, JAW, **SO**, and BEH conceived and designed the experiments. MS, YW, SJB, TRM, AS, SS, and AH performed the experiments and acquired the data. MS, YW, SJB, TRM, AS, SS, AH, JAW, and BEH analyzed and interpreted the data. MS, SJB, JAW, **SO**, and BEH wrote the paper.

All authors agree to this correction and confirm these errors do not affect the conclusions presented in the article.

We apologize for this error.

