# Supplementary material for: Inhibition of autophagy as a novel treatment for neurofibromatosis type 1 tumors
Source: Mol Oncol. 2024 Aug 11;19(3):825–51. doi: 10.1002/1878-0261.13704 (PMC11887668; doi:10.1002/1878-0261.13704)

Figure S1. Identification of synthetic lethal interactions with *NF1*.
(A) Using the dNF1-KO cells, we used a genome-wide dsRNA library to screen approximately 10,000 genes, resulting in 134 genes identified as having a synthetic lethal interaction with NF1. **(B)** Summary of results from the synthetic lethal screen. Z-scores are plotted for dNF1-KO cells on the vertical axis and WT S2R+ cells on the horizontal axis. The red box indicates the position of reagents selected as hits. **(C)** Correlation analysis of screen replicates indicating high data quality. Each bar represents the correlation coefficient from comparison between two screen replicates in WT or dNF1-KO cells as indicated or between the median Z-scores for each cell line. Bars indicate the mean from each pairwise combination of replicates and error bars indicate standard deviation. Figure S1A made using biorender.com.

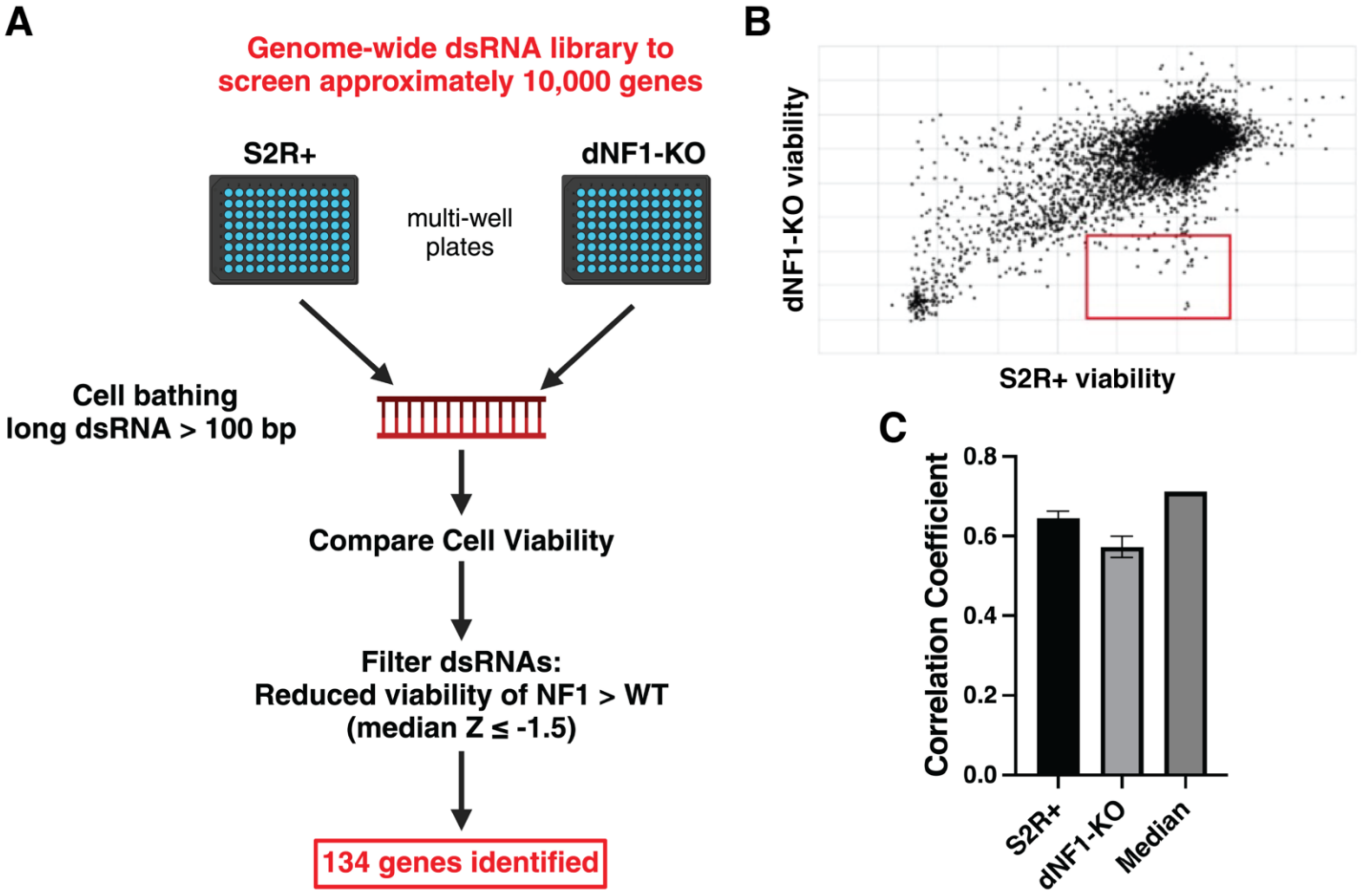

Figure S2. The NF1 gene is well conserved between Drosophila and humans with 68% identity at the amino acid level.

Alignment of amino acid sequences of neurofibromin from human (variant 2; NM_000267.3) and *Drosophila* (PD isoform, Flybase ID: FBpp0100151) using Clustal Omega (Madeira F, Pearce M, Tivey ARN, *et al.* Search and sequence analysis tools services from EMBL-EBI in 2022. Nucleic Acids Research. 2022 Jul;50(W1):W276-W279. DOI: 10.1093/nar/gkac240. PMID: 35412617; PMCID: PMC9252731).

* indicates identical amino acids, ":" conserved and "." semi-conserved residues. Boxed region indicates the GAP-related domain (GRD).

```
Dros            -MTQKPGEWASALLARFEDQLPNRIGAYGTQARMSQDQLVACLIHISRYRFSLVISGLTK 59
Human           MAAHRPVEWVQAVVSRFDEQLPIKTGQQNTHTKVSTEHNKECLINISKYKFSLVISGLTT 60
                  :::* **..*:::**::*** : *  .*::::* ::   ***:**:*:*********.

Dros            MLQRVNEAALQNRHEPERCYFESLVIILTTLERCLTNQTKDTARFEEAMNVKLLLREISQ 119
Human           ILKNVNNMRIFGEAAEKN-LYLSQLIILDTLEKCLAGQPKDTMRLDETMLVKQLLPEICH 119
                :*:.**:  : ..   :.  : * :*** ***:**:.* *** *::*:* ** ** **.:

Dros            FVDVQSDSNPNAAQLKALASKVLFALSQNHFSAVFNRISARIQELTSCSEENPDYNDIEL 179
Human           FLHTCREGNQHAAELRNSASGVLFSLSCNNFNAVFSRISTRLQELTVCSEDNVDVHDIEL 179
                *:..  :.* :**:*:  ** ***:** *:*.***.***:*:**** ***:* * :****

Dros            IQHIDMDMIKLTKLLQETITKFRSK-RAPPLILLYSLEKAIWNWIEYHPQEFQDLQRGTN 238
Human           LQYINVDCAKLKRLLKETAFKFKALKKVAQLAVINSLEKAFWNWVENYPDEFTKLYQIPQ 239
                :*:*::*  **.:**:**  **::  :.  * :: *****:***:* :*:** .* :  :

Dros            RDISTCWEPLMDFVEYFKTENKKSKTLVWPLQMLLLILNPSCLEAVVNELQQSEKEKEKD 298
Human           TDMAECAEKLFDLVDGFAES-TKRKAAVWPLQIILLILCPEIIQDISKDV---------- 288
                 *:: * * *:*:*: *  . .* *: *****::**** *. :: : :::

Dros            KEKVASKSAQSTSRDKDFSAKQFIESIKRGLGQHSPSKQVTESAAIACVKLCKASTYINN 358
Human           ------------VDENNMNKKLFLDSLRKALAGHGGSRQLTESAAIACVKLCKASTYINW 336
                            ::::. * *::*:::.*. *. *:*:******************

Dros            TDSNNVVFKLVQFFINDLKALLFNPAKPFSRGQGYNFADIELMIDCWVSCFRINPHNIEA 418
Human           ED-NSVIFLLVQSMVVDLKNLLFNPSKPFSRGSQ--PADVDLMIDCLVSCFRISPHNNQH 393
                 * *.*:* *** :: *** *****:******.    **::***** ******.*** :

Dros            LKVCLNLSSPQAYHFVIVCSLLRLAHIYVDFRLQNKNPFRIVNQPRLSWWPQTDVVHYRS 478
Human           FKICLAQNSPSTFHYVLVNSLH-----------------RIITNSALDWWPKIDAVYCHS 436
                :*:**  .**.::*:*:* **                 **:.:  *.***: *.*: :*

Dros            AELRALFTDTLNKATQGYIAHTPLRYITSLTLKSKDTQKGLTR---AEEGPAHKMLLLLL 535
Human           VELRNMFGETLHKAVQGCGAHPAIRMAPSLTFKEKVTSLKFKEKPTDLETRSYKYLLLSM 496
                .*** :* :**:**.**  **  :*   ***:*.* *. :..     *  ::* *** :

Dros            VRLIHADPTLLLNTQGKVAHEVQSSTLELINGLVSLVHQTTMPDVAQEAMEALLALHAPE 595
Human           VKLIHADPKLLLCNPRKQGPETQGSTAELITGLVQLVPQSHMPEIAQEAMEALLVLHQLD 556
                *:******.*** .  * . *.*.** ***.***.** *: **:**********.**  :

Dros            KIEVWNPEAPINTFWDVSSQVLFSISQKLIQHQIANYTDVLKWLREILICRNTFLQRHKD 655
Human           SIDLWNPDAPVETFWEISSQMLFYICKKLTSHQMLSSTEILKWLREILICRNKFLLKNKQ 616
                .*::***:**::***::***:** *.:** .**: . *::***********.** ::*:

Dros            YAH-----------------------------------------------------VGSQ 662
Human           ADRSSCHFLLFYGVGCDIPSSGNTSQMSMDHEELLRTPGASLRKGKGNSSMDSAAGCSGT 676
                  :                                                     ..

Dros            IAICKQAHIKMEVVFFMYLWSVDLDAVLTSLSCFGLLCEEAEICCSSDELTVGFIMPNYH 722
Human           PPICRQAQTKLEVALYMFLWNPDTEAVLVAMSCFRHLCEEADIRCGVDEVSVHNLLPNYN 736
                  **:**: *:**.::*:**. * :***.::***  *****:* *. **::*  ::***:

Dros            IYQELAQLSTSATDSRICFFDNTHGNVLSRLTLQKRIMTLLRKIEHCVHGVQPAWEETFR 782
Human           TFMEFASVSNMMS--------------TGRAALQKRVMALLRRIEHPTAGNTEAWEDTHA 782
                 : *:*.:*.  :              .* :****:*:***:*** . *   ***:*.

Dros            NWEVSSKVLQTYPKCKGEDGQA-EVFHRGMGKRRASHQSSEHD----LEEQINEWANMTW 837
Human           KWEQATKLILNYPKAKMEDGQAAESLHKTIVKRRMSHVSGGGSIDLSDTDSLQEWINMTG 842
                :** ::*:: .***.* ***** * :*: : *** ** *.  .       :.::** ***

Dros            FLLALGGVCLHKRSSSRQMLLQQSQNNASLGSLAQNSLYSSSTSSGHGSLHPSTVSLSTL 897
Human           FLCALGGVCLQQRSNSGLATYSPP-----MGPVS----------ERKGSM---------I 878
                ** *******::**.*     .       :* ::          . :**:         :
```

```
Dros     PPAPPQDVSYCPVTQFVGQLLRLLVCSNEKIGLNIQKNVKELVGEEMSTQLYPILFDQVR 957
Human    SVMSSEGNADTPVSKFMDRLLSLMVCNHEKVGLQIRTNVKDLVGLELSPALYPMLFNKLK 938
              :. :  **::*:.:** *:**.:**:**:*:.***:*** *:*  ***:**::::

Dros     AIVEKFFDQQGQVNVNVTDINTQFIEHTIYIMKSILDPKANKDPNNDQPSPSEHLGVTSI 1017
Human    NTISKFFDSQGQVL--LTDTNTQFVEQTIAIMKNLLDNH--------TEGSSEHLGQASI 988
           :.****.****   :** ****:*:** ***.:** :          . ***** :**

Dros     EGMMLGIVRYVRHLDMTVYAIRIKTKLCQLVEVMMKRRDDLAFRQEMKFRNKLVEYLTDW 1077
Human    ETMMLNLVRYVRVLGNMVHAIQIKTKLCQLVEVMMARRDDLSFCQEMKFRNKMVEYLTDW 1048
         * ***.:***** *.  *:**:************* *****:* ********:*******

Dros     VMGTSHQIAPPSSADAAILTNTSLIFRDLDQACMEAVAALLRGLPLQPEESDRGDLMDAK 1137
Human    VMGTSNQAADD---------DVKCLTRDLDQASMEAVVSLLAGLPLQPEEGDGVELMEAK 1099
         *****:* *           :.. : ******.****.:** ********.*  :**:**

Dros     SALFLKYFTLFMNLLNDCIDSSEAEKEMNNTPLLPPRPRMAAGKLTALRNATILAMSNLL 1197
Human    SQLFLKYFTLFMNLLNDCSEVEDESAQT------GGRKRGMSRRLASLRHCTVLAMSNLL 1153
         * **************** : .: . :         * *  : :*::**:.*:*******

Dros     GANIDSGLMHSIDLGYNPDLQTRAAFMEVLTQILQQGTEFDTLAETVLADRFEQLVQLVT 1257
Human    NANVDSGLMHSIGLGYHKDLQTRATFMEVLTKILQQGTEFDTLAETVLADRFERLVELVT 1213
         .**:********.***: ******:******:*********************:**:***

Dros     MISDKGELPIAMALANVVTTSQMDELARVLVTLFDAKHLLSPLLWNMFYREVEVSDCMQT 1317
Human    MMGDQGELPIAMALANVVPCSQWDELARVLVTLFDSRHLLYQLLWNMFSKEVELADSMQT 1273
         *:.*:*************  ** ************::***  ****** :***::*.***

Dros     LFRGNSLGSKIMAFCFKIYGASYLQMLLEPLIRPLLDEE--EETCFEVDPARLDPTEDIE 1375
Human    LFRGNSLASKIMTFCFKVYGATYLQKLLDPLLRIVITSSDWQHVSFEVDPTRLEPSESLE 1333
         *******.****:****:***:*** **:**:* :: ..  :...*****:**:*:*.:*

Dros     QHRNNLIALTQKVFDAIINSSDRFPPQLRSMCHCLYQVLSKRFPNLLQNNIGAVGTVIFL 1435
Human    ENQRNLLQMTEKFFHAIISSSSEFPPQLRSVCHCLYQVVSQRFP---QNSIGAVGSAMFL 1390
         :::.**: :*:*.*.***.**..*******:*******:*:***   **.*****:.:**

Dros     RFINPAIVSPQELGIVDKQVHSSAKRGLMLMSKILQNIANHVEFSKEQHMLCFNDFLRDH 1495
Human    RFINPAIVSPYEAGILDKKPPPRIERGLKLMSKILQSIANHVLFTKEEHMRPFNDFVKSN 1450
         ********** * **:**:     :*** *******.***** *:**:**  ****::.:

Dros     FEAGRRFFIQIASDCETVDQTSHSMSFISDANVLALHRLLWTHQEKIGDYLSSSRDHKAV 1555
Human    FDAARRFFLDIASDCPTSDAVNHSLSFISDGNVLALHRLLWNNQEKIGQYLSSNRDHKAV 1510
         *:*.****::***** * * ..**:*****.**********.:*****:****.******

Dros     GRRPFDKMATLLAYLGPPEHKPVDSHMMFSSYARWSSIDMSSTNFEEIMVKHQMHEKEEF 1615
Human    GRRPFDKMATLLAYLGPPEHKPVAD-------THWSSLNLTSSKFEEFMTRHQVHEKEEF 1563
         *********************** .       ::***:::*::***:*.:**:******

Dros     KTLKSMNIFYQAGTSKSGYPVFYYIARRYKIGETNGDLLIYHVILTLKPFCHSPFEVVID 1675
Human    KALKTLSIFYQAGTSKAGNPIFYYVARRFKTGQINGDLLIYHVLLTLKPYYAKPYEIVVD 1623
         *:**::.*********:* *:***:***:* *: *********:*****:  .*:*:*:*

Dros     FTHTCSDNRFRTEFLQKWFYVLPTVAYENVHAVYIYNCNSWVREYTKFHDRILAPLKGNR 1735
Human    LTHTGPSNRFKTDFLSKWFVVFPGFAYDNVSAVYIYNCNSWVREYTKYHERLLTGLKGSK 1683
         :***  .***:*:**.*** *:* .**:** ****************:*:*:*: ***.:

Dros     KLLFLESPNKLTDFIDAEQQKLPGATLSLDEDLKVFSNALKLSHKDTKVAIKVGPTALQI 1795
Human    RLVFIDCPGKLAEHIEHEQQKLPAATLALEEDLKVFHNALKLAHKDTKVSIKVGSTAVQV 1743
         :*:*::.*.**::.*: ******.***:*:****** *****:******:**** **:*:

Dros     TSAEKTKVLAHSVLLNDVYYASEIEEVCLVDDNQFTLSITNESGQLSFIHNDCDNIVQAI 1855
Human    TSAERTKVLGQSVFLNDIYYASEIEEICLVDENQFTLTIANQGTPLTFMHQECEAIVQSI 1803
         ****:****.:**:***:********:****:*****:*:*:.  *:*:*::*: ***:*
```

```
Dros       IHIRNRWELSQPDSVTVHQKIRPKDVPGTLLNMALLNLGSCDPNLRTAAYNLLCALTATF 1915
Human      IHIRTRWELSQPDSIPQHTKIRPKDVPGTLLNIALLNLGSSDPSLRSAAYNLLCALTCTF 1863
           ****.*********:  * *************:*******.**.**:**********.**

Dros       DLKIEGQLLETQGLCIPSNNTIFIKSVSEKLATNEPHLTLEFLEESIQGFQRSTIELKHL 1975
Human      NLKIEGQLLETSGLCIPANNTLFIVSISKTLAANEPHLTLEFLEECISGFSKSSIELKHL 1923
           :**********.*****:***:** *:*:.**:************.*.**.:*:******

Dros       CLEYMTPWLKNLVKFCKSNDDSKKLKVSQILDKLINLTIDQKEMYPSVQAKIWGSIGQIP 2035
Human      CLEYMTPWLSNLVRFCKHNDDAKRQRVTAILDKLITMTINEKQMYPSIQAKIWGSLGQIT 1983
           *********.***:*** ***:*: :*: ******.:**::*:****:*******:***

Dros       ELIDMVLDNFLHKSITYGLGSPQVEIMADTAVALASANVQLVSKKVITRICRVMDKSCTN 2095
Human      DLLDVVLDSFIKTSATGGLGSIKAEVMADTAVALASGNVKLVSSKVIGRMCKIIDKTCLS 2043
           :*:*:***.*::.* * **** :.*:**********.**:***.*** *:*:::**:* .

Dros       PTQYLEQHMMWDDIAILGRYLLMLSFNNCLDVATSVPYLFHTITFLVCSGSLSMRASTHG 2155
Human      PTPTLEQHLMWDDIAILARYMLMLSFNNSLDVAAHLPYLFHVVTFLVATGPLSLRASTHG 2103
           **  ****:********.**:*******.****: :*****.:****.:* **:******

Dros       LVINIIHSLCTCTNPSFSEEAQRVLRLSLDEFSLPKFYLLFGISKVKSAAVTAFRSSCRH 2215
Human      LVINIIHSLCTCSQLHFSEETKQVLRLSLTEFSLPKFYLLFGISKVKSAAVIAFRSSYRD 2163
           ************::  ****::****** ******************** ***** *.

Dros       PTDKWLGNERVTQPLPADRERLSLPSLEVITDALLEIMEACMRDVPDCEWLNTWTSLARS 2275
Human      R---------SFSPGSYERETFALTSLETVTEALLEIMEACMRDIPTCKWLDQWTELAQR 2214
                     .*   :** ::* ***.:*:************:* *:**: **.**:

Dros       FAFCYNPALQPRALIVYGCISKSVTDHEVKQLLRILVKALE-------SFNDLILIEALV 2328
Human      FAFQYNPSLQPRALVVFGCISKRVSHGQIKQIIRILSKALESCLKGPDTYNSQVLIEATV 2274
           *** ***:******:*:***** *:. ::**::*** ****       ::*. :**** *

Dros       MCLTRIQPLLRPESPIHRALFWVAISVLQLDEITLYGAGLALLEQNLHTLKSQGCFDKKE 2388
Human      IALTKLQPLLNKDSPLHKALFWVAVAVLQLDEVNLYSAGTALLEQNLHTLDSLRIFNDKS 2334
           :.**::****. :**:*:******::******:.**.** **********.*   *:.*.

Dros       TIAEVMMKTREKLEWHFKQLDHAVGLSFRSNFHFALVGHLIKGFRHPTPTTVSRTSRVLT 2448
Human      -PEEVFMAIRNPLEWHCKQMDHFVGLNFNSNFNFALVGHLLKGYRHPSPAIVARTVRILH 2393
              **:*  *: **** **:** ***.*.***:*******:**:***:*: *:** *:*

Dros       MLLGIIAKPLHRDKFEVTPDSVAYLTALVAVSEEVRSRCHVKHALPRWPADLSSSV---- 2504
Human      TLLTLVNKHRNCDKFEVNTQSVAYLAALLTVSEEVRSRCSLKHRKSLLLTDISMENVPMD 2453
            ** :: *  : *****. :*****:**::********* :**      :*:* .

Dros       ----ENGEASGGVQAIGLPLSRRQKSWDILDQSALQ-----------------FARQHK 2542
Human      TYPIHHGDPSYRT-------LKETQPWSSPKGSEGYLAATYPTVGQTSPRARKSMSLDMG 2506
               .:*: *  .        :. : *.  . *                    :: :

Dros       VPTLQNARVLFKTQRSFSVPT--TKDPN---NATGI---------------------EE 2575
Human      QPSQANTKKLLGTRKSFDHLISDTKAPKRQEMESGITTPPKMRRVAETDYEMETQRISSS 2566
            *:  *:: *: *::**.     ** *:     :**                       ..

Dros       RQERGSRSSVSNESNVLLDPEVLPDLSIQALVLTVLATLVKYSSDEGETRVLYQYLAEGS 2635
Human      QQHPHLRKVSVSESNVLLDEEVLTDPKIQALLLTVLATLVKYTTDEFDQRILYEYLAEAS 2626
           :*.   *.   .******* *** * .****:**********::** : *:**:****.*

Dros       VVFPKVFPVIHSLLDQKINNILSVSHDQVVLNSVQNIIQNMLA-SEDPSQQQLHFLQSCG 2694
Human      VVFPKVFPVVHNLLDSKINTLLSLCQDPNLLNPIHGIVQSVVYHEESPPQYQTSYLQSFG 2686
           *********:*.***.***.:**:.:*  :** ::.*:*.::  .*.* * *  :*** *

Dros       FGGLWRFAGPFTKYNMMGESSELFVNCLEAMVETCLPGDESAP------VPPSPRPYNLS 2748
Human      FNGLWRFAGPFSKQTQIPDYAELIVKFLDALIDTYLPGIDEETSEESLLTPTSPYPPALQ 2746
           *.*********:* . : : :**:*: *:*:::* *** :.        .* ** *  *.

Dros       SSLSSL---TLGSPTD------------KAA---------------------------- 2764
Human      SQLSITANLNLSNSMTSLATSQHSPGIDKENVELSPTTGHCNSGRTRHGSASQVQKQRSA 2806
           *.**     .*..               *

Dros       ------------ 2764
Human      GSFKRNSIKKIV 2818
```

Figure S3. Western blot data

(A) Relates to Figure 1B. **(B)** Relates to Figure 1B-C. **(C-D)** Relate to Figure 1F-G. **(E-F)** Further assessment of pERK1 and pAKT under complete media (CM) and serum free (SF) conditions in S2R+/dNF1-KO cells **(E)** and WT/C8/C23 Schwann cells **(F)**. Each lane represents a single repeat.

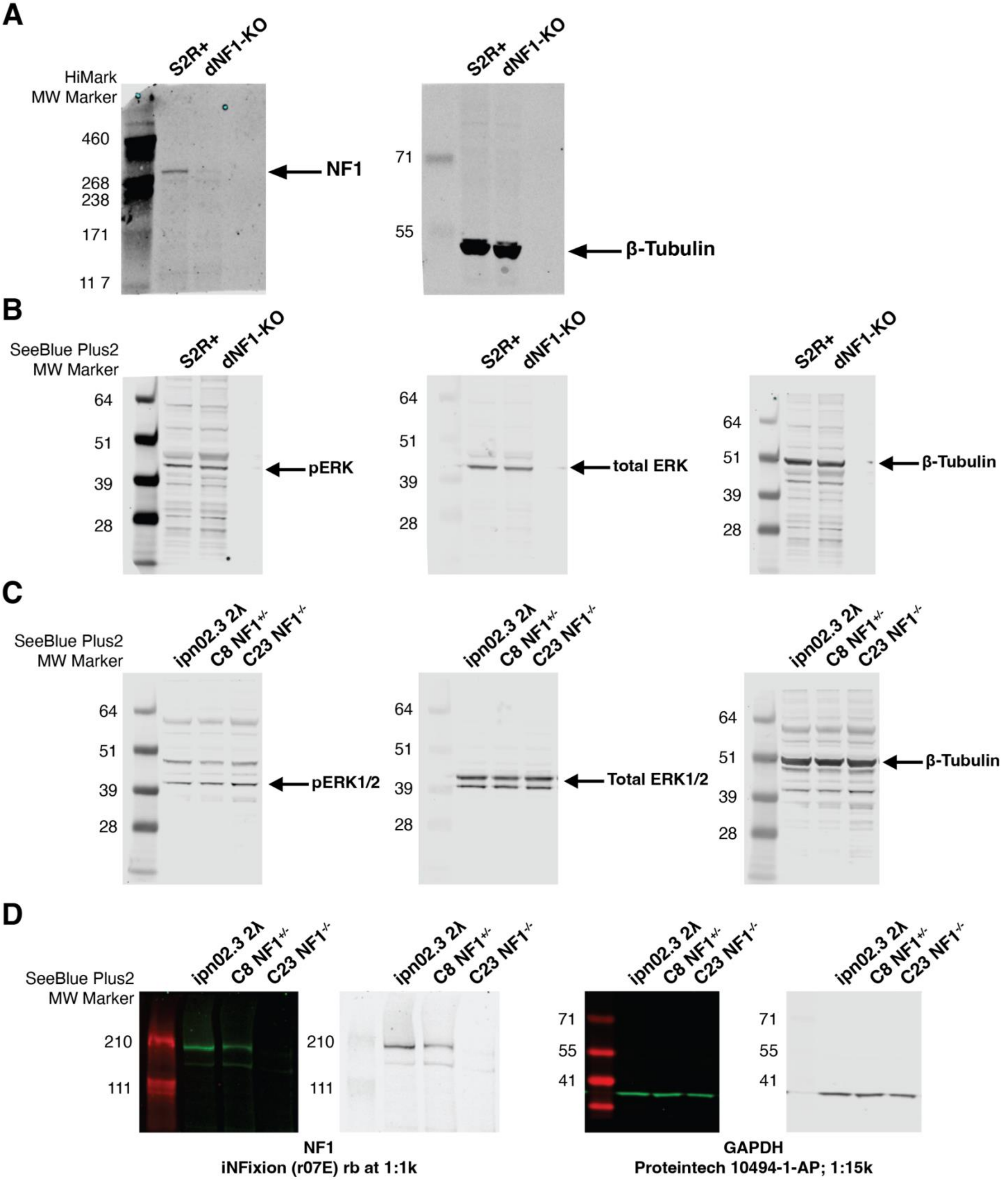

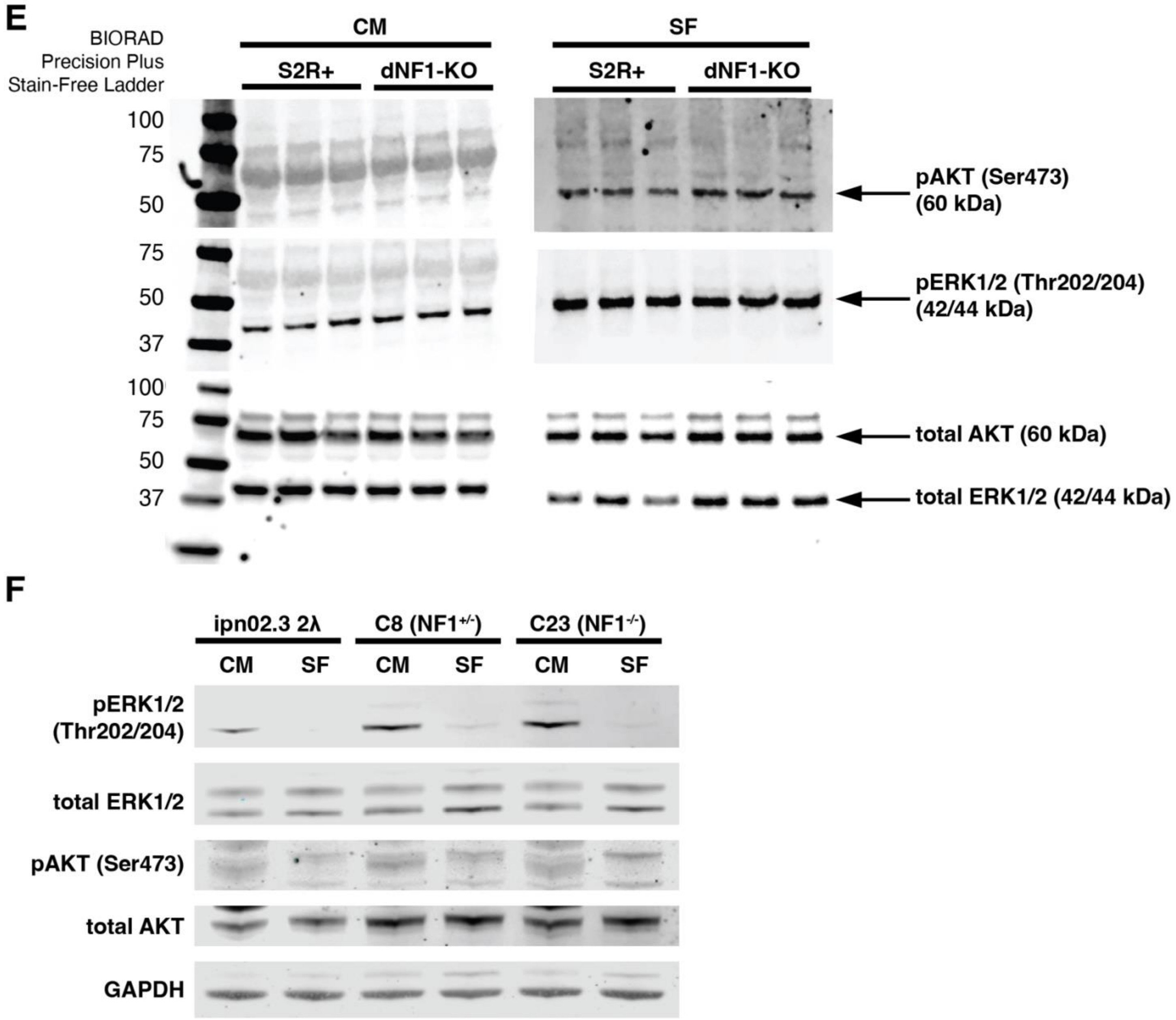
E
BIORAD
Precision Plus
Stain-Free Ladder
CM
SF
S2R+
dNF1-KO
S2R+
dNF1-KO
100
75
50
75
50
37
100
75
50
37
pAKT (Ser473)
(60 kDa)
pERK1/2 (Thr202/204)
(42/44 kDa)
total AKT (60 kDa)
total ERK1/2 (42/44 kDa)
F
ipn02.3 2λ
C8 (NF1+/-)
C23 (NF1-/-)
CM
SF
CM
SF
CM
SF
pERK1/2
(Thr202/204)
total ERK1/2
pAKT (Ser473)
total AKT
GAPDH

Figure S4. Drug dose curves in Drosophila cells.
(A) Cell-Titer Glo dose curves for the seven drugs with *Drosophila* orthologs as targets in S2R+ and dNF1-KO cells. **(B)** Dose curves for CQ and Bafilomycin A1 in human CRISPR/Cas9 genome edited Schwann cells ($NF1^{+/-}$ and $NF1^{-/-}$). **(C)** Dose curves for CQ and Bafilomycin A1 in human patient cells: ipnNF95.11C ($NF1^{+/-}$) and ipNF95.11b 'C' ($NF1^{-/-}$). **(C)** Cell counts in human WT Schwann cells following treatment with CQ and Bafilomycin A1. **(D)** Cell-Titer Glo assay comparing the effects of CQ and Selumetinib in C8 ($NF1^{+/-}$) and C23 ($NF1^{-/-}$) cells. All error bars represent the standard deviation.

A

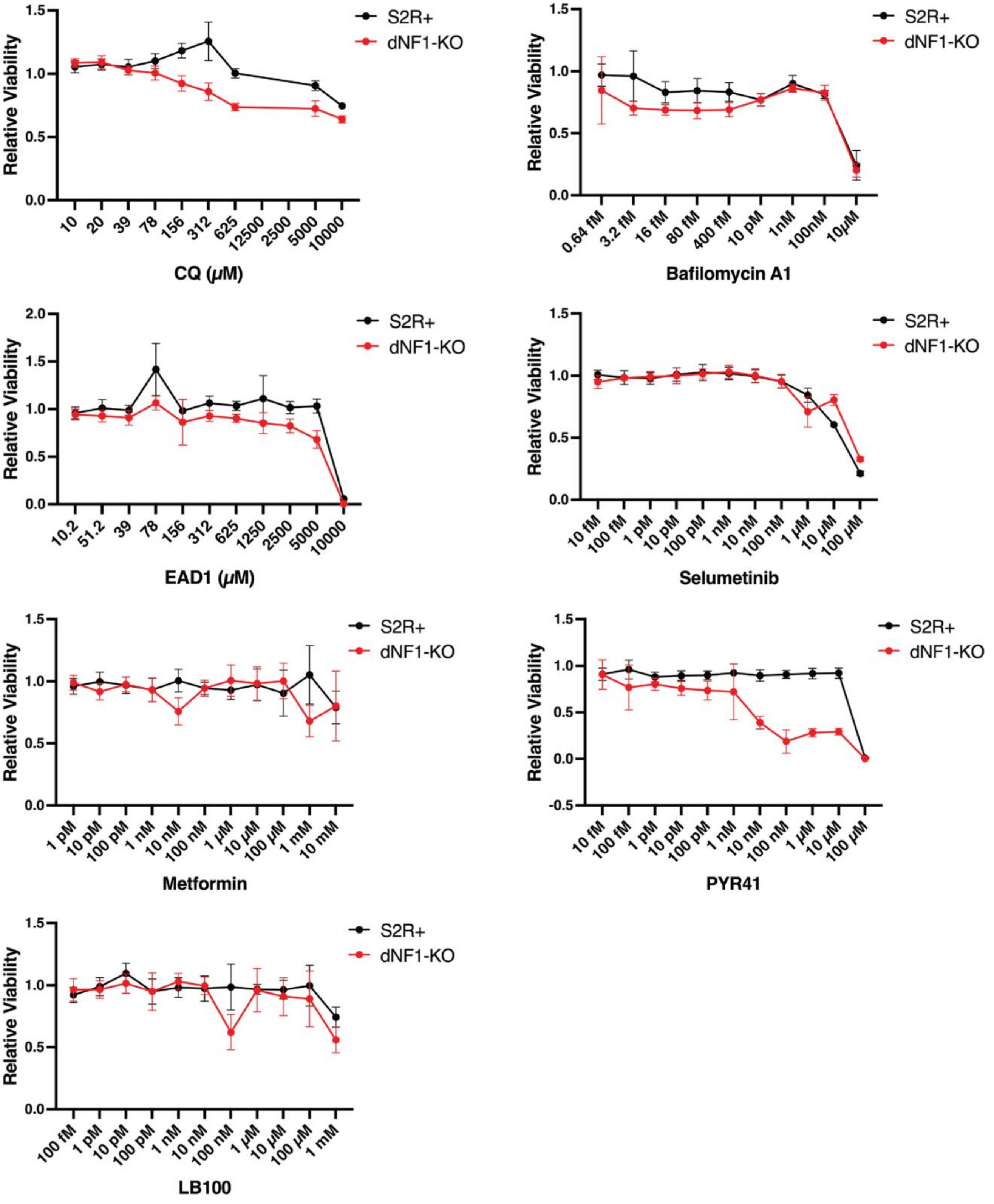

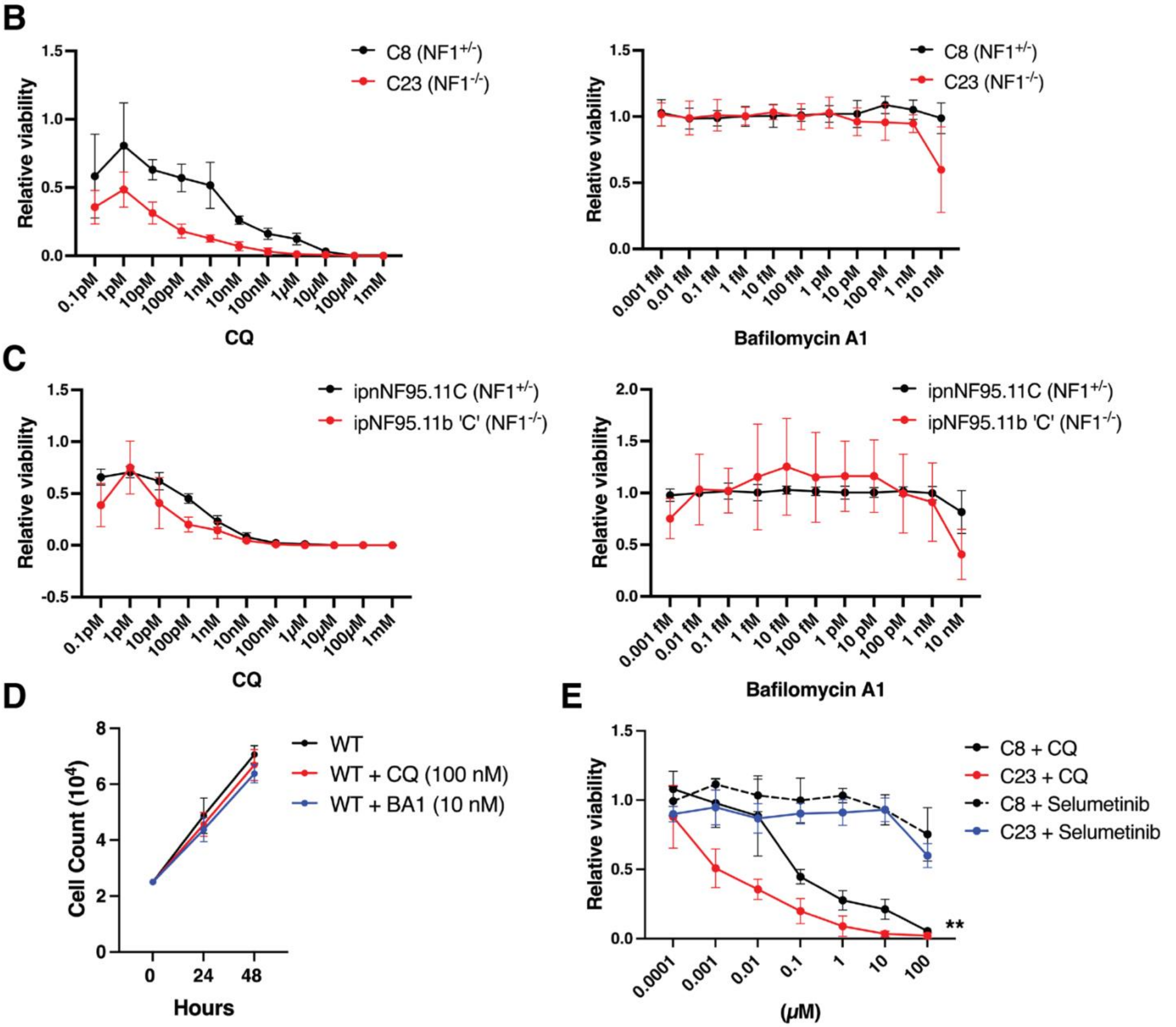
B
C8 (NF1+/-)
C23 (NF1-/-)
Relative viability
CQ
Bafilomycin A1
C
ipnNF95.11C (NF1+/-)
ipNF95.11b 'C' (NF1-/-)
Relative viability
CQ
Bafilomycin A1
D
WT
WT + CQ (100 nM)
WT + BA1 (10 nM)
Cell Count (10^4)
Hours
E
C8 + CQ
C23 + CQ
C8 + Selumetinib
C23 + Selumetinib
Relative viability
(μM)
**

Figure S5. Autophagy pathway activity in NF1-deficient and control cells.
(A) Live lysosome activity assay. ipnNF9511.C and ipNF95.11b 'C' cells were treated with/without CQ or bafilomycin A1 (BA1) for 2 h in serum free media before measuring lysosomal activity using a lysosome-specific self-quenching substrate (abcam). The substrate acts as endocytic cargo, and upon degradation, a fluorescent signal is generated. Therefore, fluorescence is proportional to lysosome activity. Increases fluorescence (green) was observed in ipNF95.11b 'C' ($NF1^{-/-}$) cells compared to heterozygous controls. CQ and BA1 inhibited lysosomal activity in both cells. Nuclei are shown in blue (marked with DAPI). Scale bar = 250 μM. **(B)** LC3-B western blot. Baseline levels of autophagy were found to be higher in ipNF95.11b 'C' ($NF1^{-/-}$) cells compared to ipnNF95.11C ($NF1^{+/-}$) cells (*P<0.05). Each lane represents a single repeat. LC3-II normalised to total protein imaged on BIORAD TGX stain free gels. Error bars represent the standard deviation.

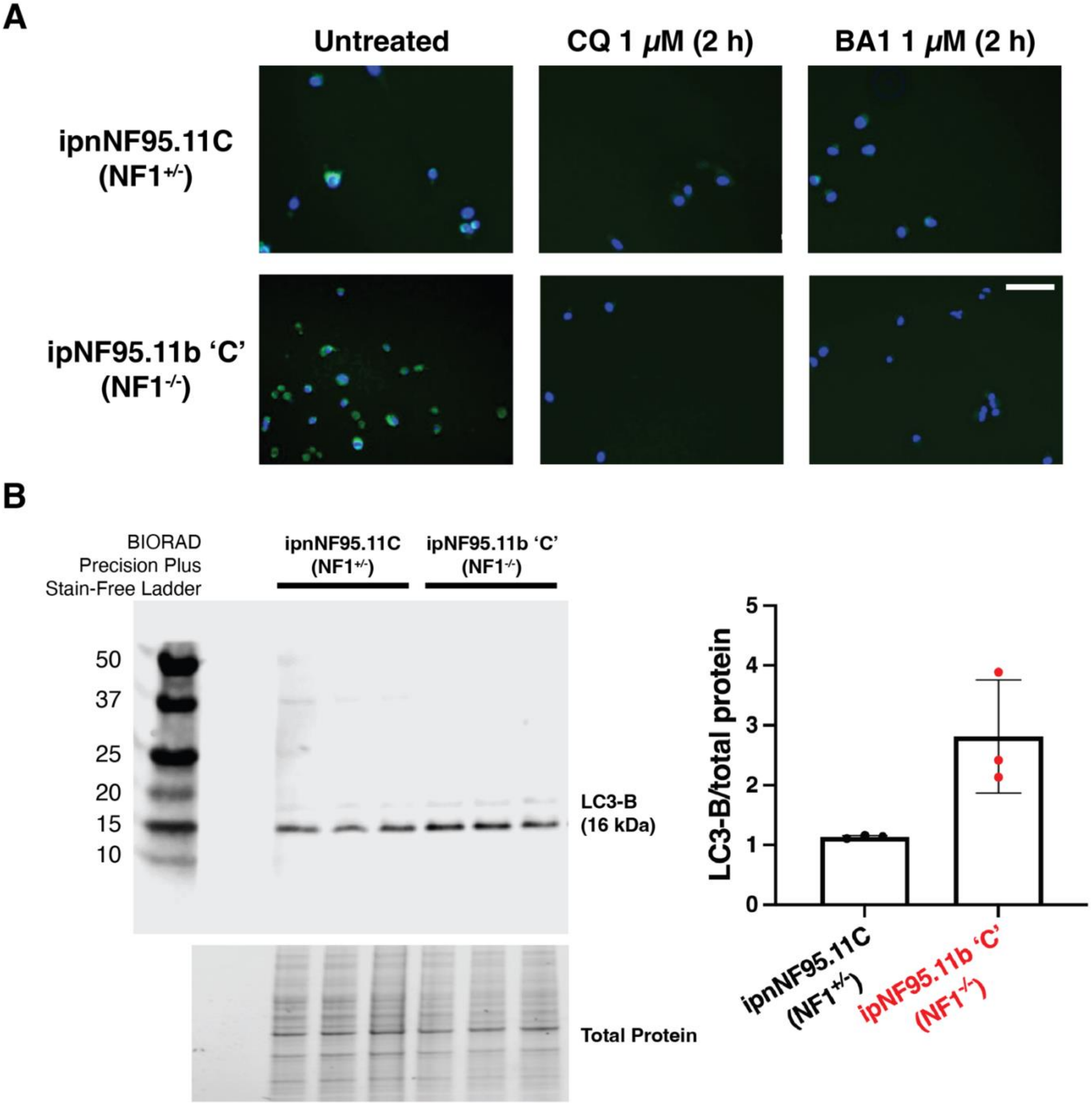

Figure S6. Drug dose curves of autophagy inhibitors in human cells.
Dose curves for the five autophagy drugs in patient cells: ipnNF95.11C (NF1$^{+/-}$) and ipNF95.11b ‘C’ (NF1$^{-/-}$), and ipnNF09.4 (NF1$^{+/-}$) and ipNF05.5 (NF1$^{-/-}$). Error bars represent the standard deviation.

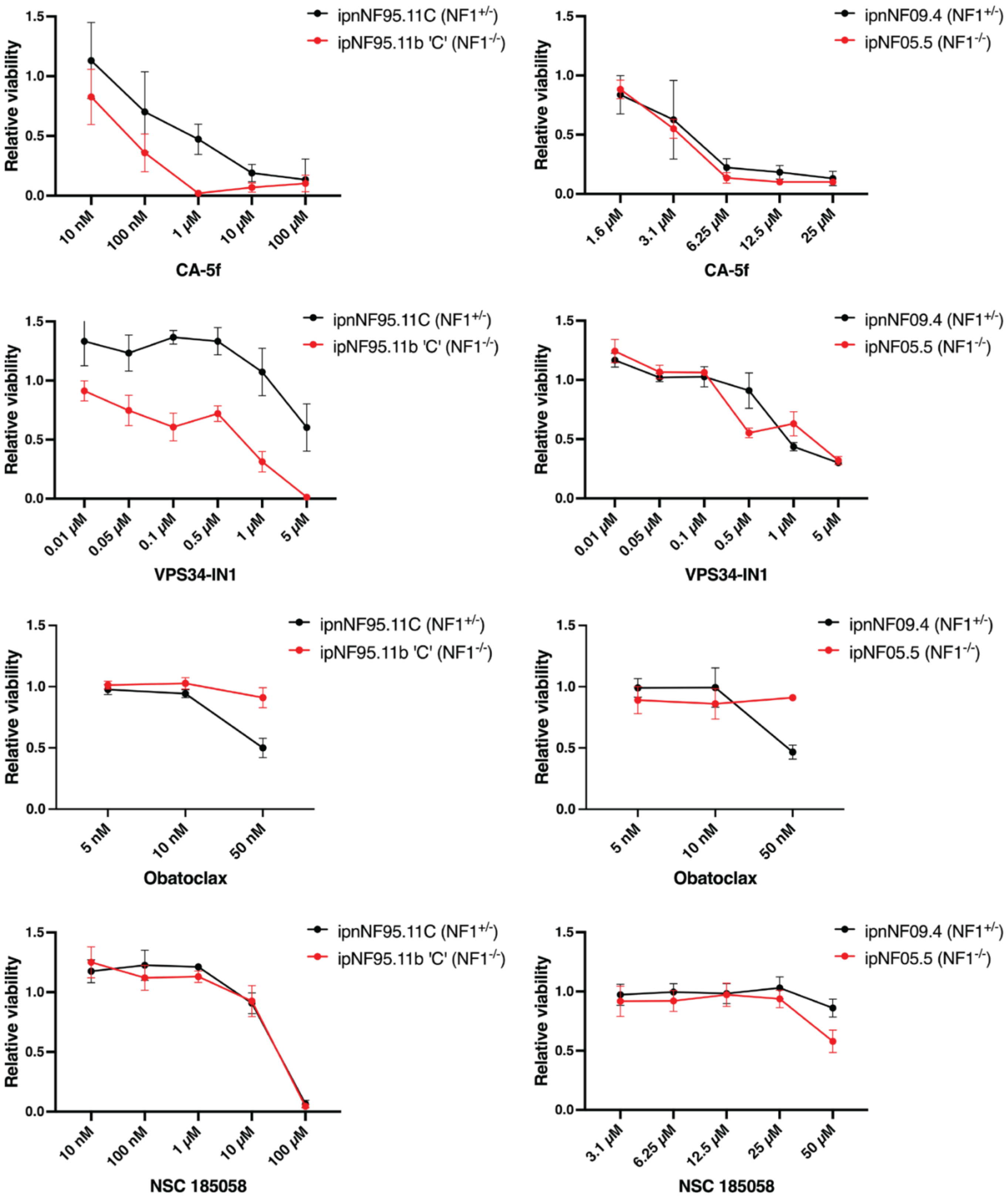

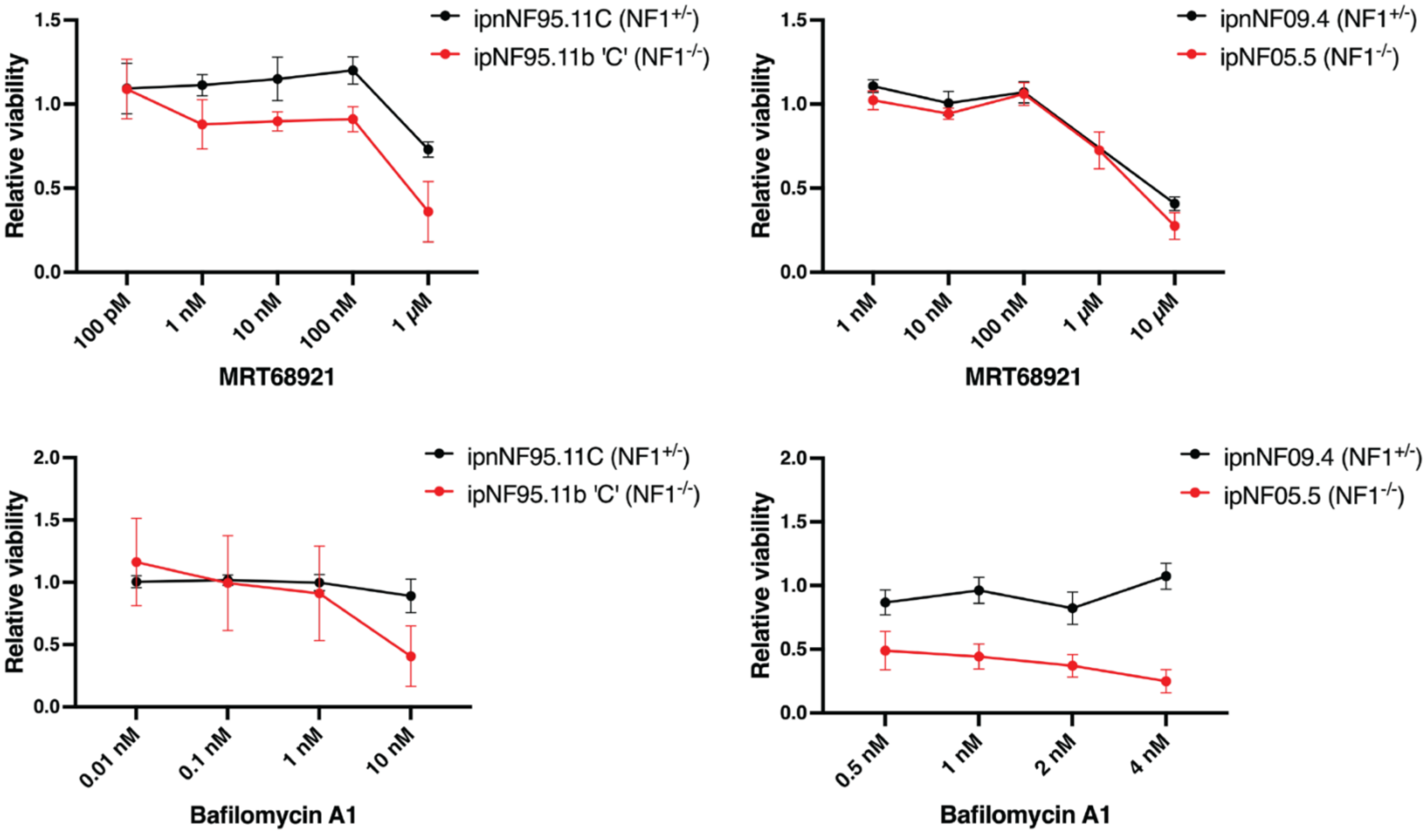
ipnNF95.11C (NF1$^{+/-}$)
ipNF95.11b 'C' (NF1$^{-/-}$)
Relative viability
0.0
0.5
1.0
1.5
100 pM
1 nM
10 nM
100 nM
1 μM
MRT68921
ipnNF09.4 (NF1$^{+/-}$)
ipNF05.5 (NF1$^{-/-}$)
Relative viability
0.0
0.5
1.0
1.5
1 nM
10 nM
100 nM
1 μM
10 μM
MRT68921
ipnNF95.11C (NF1$^{+/-}$)
ipNF95.11b 'C' (NF1$^{-/-}$)
Relative viability
0.0
0.5
1.0
1.5
2.0
0.01 nM
0.1 nM
1 nM
10 nM
Bafilomycin A1
ipnNF09.4 (NF1$^{+/-}$)
ipNF05.5 (NF1$^{-/-}$)
Relative viability
0.0
0.5
1.0
1.5
2.0
0.5 nM
1 nM
2 nM
4 nM
Bafilomycin A1

Figure S7. CQ results in an increased dose-dependent lethality of dNf1[C1] mutants compared to control flies. Fly food was supplemented with the indicated concentrations of CQ and survival determined by counting living flies at each time point (n=3, 20 flies per experiment). All error bars represent the standard deviation.

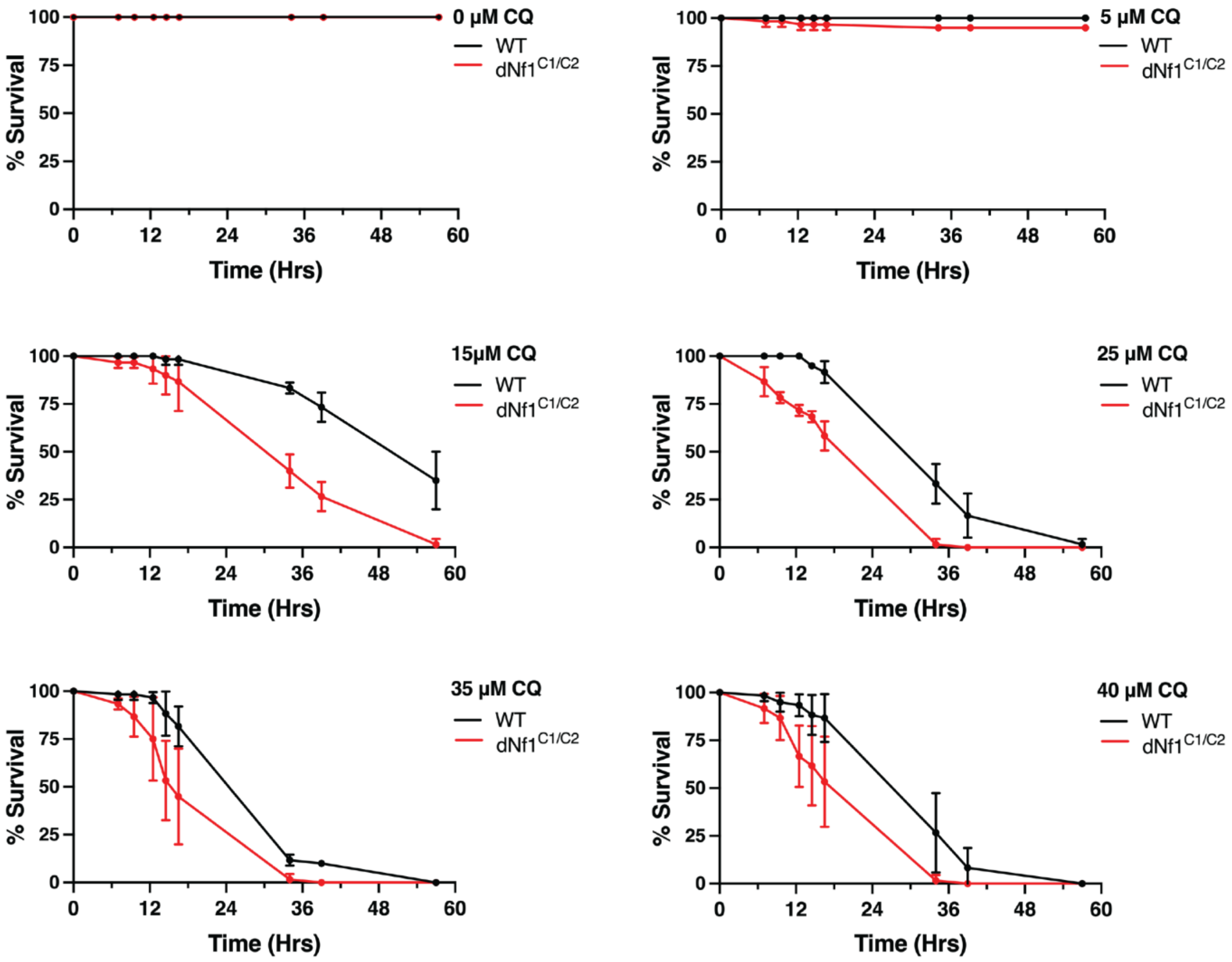

Figure S8. Mouse weight change during treatment.
The adult mice were weighted three times per week during treatment with either saline (control), CQ, or selumetinib. No mice lost weight during the treatment period, indicating no toxic effects associated with the dose used of CQ (50 mg/kg) and selumetinib (25 mg/kg). Error bars represent the standard deviation.

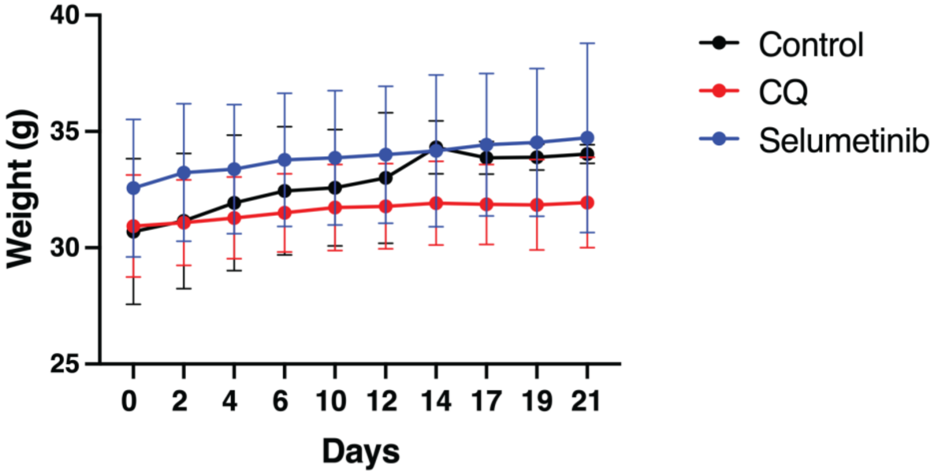

Supplement: Supplementary file 1 — Fig. S1. Identification of synthetic lethal interactions with NF1. Fig. S2. The NF1 gene is well conserved between Drosophila and humans with 68% identity at the amino acid level. Fig. S3. Western blot data. Fig. S4. Drug dose curves in Drosophila cells. Fig. S5. Autophagy pathway activity in NF1‐deficient and control cells. Fig. S6. Drug dose curves of autophagy inhibitors in human cells. Fig. S7. CQ results in an increased dose‐dependent lethality of dNf1 C1 mutants compared to WT control flies. Fig. S8. Mouse weight change during treatment. [file MOL2-19-825-s001.pdf]
